# Impact of spindle-inspired transcranial alternating current stimulation during a nap on sleep-dependent motor memory consolidation in healthy older adults

**DOI:** 10.1093/sleepadvances/zpaf022

**Published:** 2025-03-26

**Authors:** Maëva Moyne, Manon Durand-Ruel, Chang-Hyun Park, Roberto Salamanca-Giron, Virgine Sterpenich, Sophie Schwartz, Friedhelm C Hummel, Takuya Morishita

**Affiliations:** Defitech Chair of Clinical Neuroengineering, Neuro X Institute (INX), École Polytechnique Fédérale de Lausanne (EPFL), Geneva, Switzerland; Defitech Chair of Clinical Neuroengineering, INX, EPFL Valais, Clinique Romande de Réadaptation, Sion, Switzerland; Clinical Neuroscience, University of Geneva Medical School, Geneva, Switzerland; Defitech Chair of Clinical Neuroengineering, Neuro X Institute (INX), École Polytechnique Fédérale de Lausanne (EPFL), Geneva, Switzerland; Defitech Chair of Clinical Neuroengineering, INX, EPFL Valais, Clinique Romande de Réadaptation, Sion, Switzerland; Defitech Chair of Clinical Neuroengineering, Neuro X Institute (INX), École Polytechnique Fédérale de Lausanne (EPFL), Geneva, Switzerland; Defitech Chair of Clinical Neuroengineering, INX, EPFL Valais, Clinique Romande de Réadaptation, Sion, Switzerland; Defitech Chair of Clinical Neuroengineering, Neuro X Institute (INX), École Polytechnique Fédérale de Lausanne (EPFL), Geneva, Switzerland; Defitech Chair of Clinical Neuroengineering, INX, EPFL Valais, Clinique Romande de Réadaptation, Sion, Switzerland; Fondation Campus Biotech Geneva, Geneva, Switzerland; Department of Basic Neurosciences, University of Geneva Medical School, Geneva, Switzerland and; Swiss Center for Affective Sciences, University of Geneva, Geneva, Switzerland; Department of Basic Neurosciences, University of Geneva Medical School, Geneva, Switzerland and; Swiss Center for Affective Sciences, University of Geneva, Geneva, Switzerland; Defitech Chair of Clinical Neuroengineering, Neuro X Institute (INX), École Polytechnique Fédérale de Lausanne (EPFL), Geneva, Switzerland; Defitech Chair of Clinical Neuroengineering, INX, EPFL Valais, Clinique Romande de Réadaptation, Sion, Switzerland; Clinical Neuroscience, University of Geneva Medical School, Geneva, Switzerland; Defitech Chair of Clinical Neuroengineering, Neuro X Institute (INX), École Polytechnique Fédérale de Lausanne (EPFL), Geneva, Switzerland; Defitech Chair of Clinical Neuroengineering, INX, EPFL Valais, Clinique Romande de Réadaptation, Sion, Switzerland

**Keywords:** healthy aging, motor learning, nap, sleep-dependent memory consolidation, spindles, transcranial alternating current stimulation

## Abstract

With the increase in life expectancy and the rapid evolution of daily life technologies, older adults must constantly learn new skills to adapt to society. Sleep reinforces skills acquired during the day and is associated with the occurrence of specific oscillations such as spindles. However, with age, spindles deteriorate and thus likely contribute to memory impairments observed in older adults. The application of electric currents by means of transcranial alternating current stimulation (tACS) with spindle-like waveform, applied during the night, was found to enhance spindles and motor memory consolidation in young adults. Here, we tested whether tACS bursts inspired by spindles applied during daytime naps may (i) increase spindle density and (ii) foster motor memory consolidation in older adults. Twenty-six healthy older participants performed a force modulation task at 10:00, were retested at 16:30, and the day after the initial training. They had 90-minute opportunity to take a nap while verum or placebo spindle-inspired tACS bursts were applied with similar temporal parameters to those observed in young adults and independently of natural spindles, which are reduced in the elderly. We show that the density of natural spindles correlates with the magnitude of memory consolidation, thus confirming that spindles are promising physiological targets for enhancing memory consolidation in older adults. However, spindle-inspired tACS, as used in the present study, did not enhance either spindles or memory consolidation. We therefore suggest that applying tACS time-locked to natural spindles might be required to entrain them and improve their related functions.

**Statement of Significance**

In the light of increasing evidence that sleep disruption is crucially involved in the progression of aging, sleep appears to be a promising treatment target in the elderly, particularly for counteracting motor memory consolidation decline. This study demonstrates the importance of sleep spindles during a daytime nap in older adults and advances our understanding of the mechanisms of non-invasive brain stimulation. These results encourage future stimulation approaches to be tailored to natural spindles (occurrence and frequency), implying that individual sleep patterns should be closely evaluated prior to sleep interventions. The importance of spindles for motor learning that is found in this study also supports future research applying spindle-targeted stimulation in populations suffering from motor deficits, such as after a stroke.

## Introduction

Motor learning promotes the acquisition of novel skills ranging from simple to more complex motor behavior. Motor skills improve with practice [1, 2] and are further reactivated and reprocessed during sleep [3, 4], leading to additional behavioral improvement or stabilization, referred to as sleep-dependent memory consolidation [5–7]. Short sleep times, such as daytime naps, appeared to be sufficient to observe such behavioral effects [8–13] (see [14] for a meta-analysis). Sleep oscillatory features like the slow waves (SW) [15–17] and sleep spindles [18] have been suggested to favor the consolidation of memories occurring during sleep. Spindles are waxing-waning bursts of sigma (11–16 Hz) oscillatory activity lasting between 0.3 and 2 seconds during light non-rapid eye movement sleep (NREM2) and deep NREM3. They have been repeatedly found to contribute to brain plasticity [19–21] by promoting long-term potentiation (LTP) [22–25]. Their features [26–29] and pattern of occurrence [30, 31] support efficient memory reprocessing, reorganization, and consolidation of motor memory traces after a night of sleep [27, 29, 31–35] and daytime naps [8, 9, 13, 36].

With normal aging, the benefits of night sleep [37–39] or nap [37, 40, 41] on memory consolidation are diminished. This can be due to age-related sleep alterations. The sleep architecture, including a reduction in sleep time, sleep depth, and an increase in sleep fragmentation, is affected by age [42–44]. Sleep oscillations (mainly SW and spindles) are also impacted. With age, spindles are reduced in their number, duration, and frequency [45–49]. Among the small number of studies that correlated spindles with memory consolidation in older adults, some found an absence of association [40, 41], while others reported a correlation between spindles and memory in young adults [39, 50]. This observation suggests that spindle-related memory consolidation processes are not yet fully understood, and/or that these processes may show heterogeneous changes within the elderly population.

Recent work has sought to modulate sleep experimentally to enhance memory consolidation. Non-invasive methods, including acoustic [51], olfactory [52], rocking [53], transcranial magnetic stimulation (TMS) [54], and transcranial electrical stimulation (tES) approaches [55, 56], have been designed to improve memory consolidation [57]. tES methods include transcranial direct current stimulation (tDCS) and transcranial alternating current stimulation (tACS). They both apply a weak electrical current (<2 mA) to the scalp, with the possibility to create oscillating waveforms to modulate neural activity in the targeted frequency band. Marshall *et al.* [56, 58] were the first to assess the effects of anodal slow oscillatory-tDCS (so-tDCS) during a night of sleep in young adults. They observed that so-tDCS improved declarative memory, which was linked with an increase in SW activity, but motor memory was unchanged. Conversely, in older adults, so-tDCS was ineffective at boosting either type of memory when applied during a night of sleep [59, 60] and improved only declarative memory when applied during a nap [55, 61]. Thus, none of these so-tDCS studies reported enhanced consolidation of motor skills (for reviews, see [62, 63]). By contrast, spindle-like stimulation enhanced motor skill consolidation in animals [19] and in humans [64]. Lustenberger *et al.* applied spindle-like tACS across frontal and centro-parietal regions in a closed-loop manner, i.e. whenever naturally occurring spindles were detected during sleep. They found that spindle-like tACS increased spindle activity between 12 and 16 Hz during NREM2 in young adults, and that this increase correlated with motor skill improvement on a sequential finger-tapping task. These findings directly inspired the present study. Since older adults show impaired motor memory consolidation and reduced spindle activity, a spindle-inspired approach seems to be promising to boost sleep-related motor memory consolidation. Here, we tested an innovative stimulation paradigm mimicking learning-relevant features of sleep spindles (i.e. spindle-inspired tACS). We expected that (i) spindle-inspired tACS would increase the density of spindles and (ii) that it would therefore enhance motor memory consolidation in older adults.

## Methods

### Participants

Forty-three healthy older adults (27 females, mean ± SD, 69.5 ± 4.6 years) were enrolled in a randomized, double-blind, sham-controlled, parallel-design study. Four participants received a pilot/previous version of the stimulation during the nap; data of three participants were excluded because of technical problems, and 10 participants did not sleep during the afternoon break and are thus part of the no-nap group (see Supplementary Figure S1). To address the effects of spindle-inspired tACS applied during a nap, we included the 26 healthy older adults who had a nap in the afternoon (17 females, mean ± SD, 69.8 ± 4.0 years) during which 11 received sham spindle-inspired tACS (10 female, mean ± SD, 68.9 ± 4.5 years) and 15 received verum spindle-inspired tACS (seven females, mean ± SD, 70.5 ± 4.6 years). Inclusion criteria were right-handedness (verified with the Edinburgh handedness inventory [65]; mean laterality quotient: 84.8 ± 20.5) and age older than or equal to 60. The exclusion criteria were the presence of neuropsychiatric diseases, intake of psychoactive medications, known or suspected alcohol or drug abuse, contraindications for magnetic resonance imaging (MRI), and contraindications for TMS and tES. During the screening (day 0), a battery of questionnaires was filled, including the Montréal Cognitive Assessment (MoCA) [66], the Pittsburgh Sleep Quality Index (PSQI) [67], and the napping behavior questionnaire [68]. Motor function was assessed by the nine-hole peg test [69] and the maximal grip force task. The regularity of the sleep/wake rhythm of the two nights preceding day 1 was checked using actigraphy (ActiGraph wGT3X-BT, Manufacturing Technology, Inc. [MTI], Northwest Florida) and continued until the end of the experiment coupled with the consensus sleep diary [70]. The study was approved by the cantonal ethics committee in Geneva (project number: 2017-00224), and all participants provided written informed consent. The study conformed to the standards according to the Declaration of Helsinki.

### Sequential grip force modulation task

**Figure 1.**
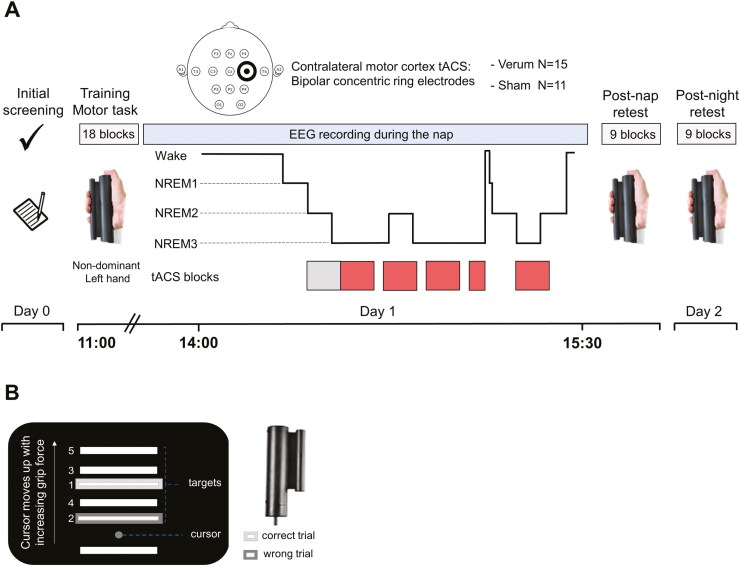
Study design and sequential grip force modulation task. (**A**) On day 0, screening was performed at least two nights before day 1. The practice of the sequential grip force modulation task consisted of 3 sessions distributed over 2 consecutive days. The morning training included two sessions. After lunch, participants were invited to take a nap under polysomnography (PSG) monitoring, during which they randomly received either the sham or verum spindle-inspired transcranial alternating current stimulation (tACS). The representation of the head shows an example of the location of the concentric ring electrodes over the right motor cortex (contralateral to the trained hand) and the 12 EEG electrodes. The spindle-inspired tACS started after 4 minutes of stable non-rapid eye movement 2 (NREM2) sleep (the leftmost rectangle at the bottom) and was applied by blocks of 4 minutes (the other rectangles at the bottom separated by gaps) separated by 2 minutes without stimulation. Participants were then retested after the nap at ~16:30 (post-nap retest), and the next day after a night of sleep (post-night retest: day 2). (**B**) The sequential grip force modulation task utilized a gripper. The visual displayed consisted of 5 targets. Participants were instructed to reach targets sequentially as fast and precisely as possible by pressing and releasing the gripper with their non-dominant left hand. Visual feedback was provided to the participants to indicate correct and incorrect trials (light and dark gray frames, respectively).

**Figure 2.**
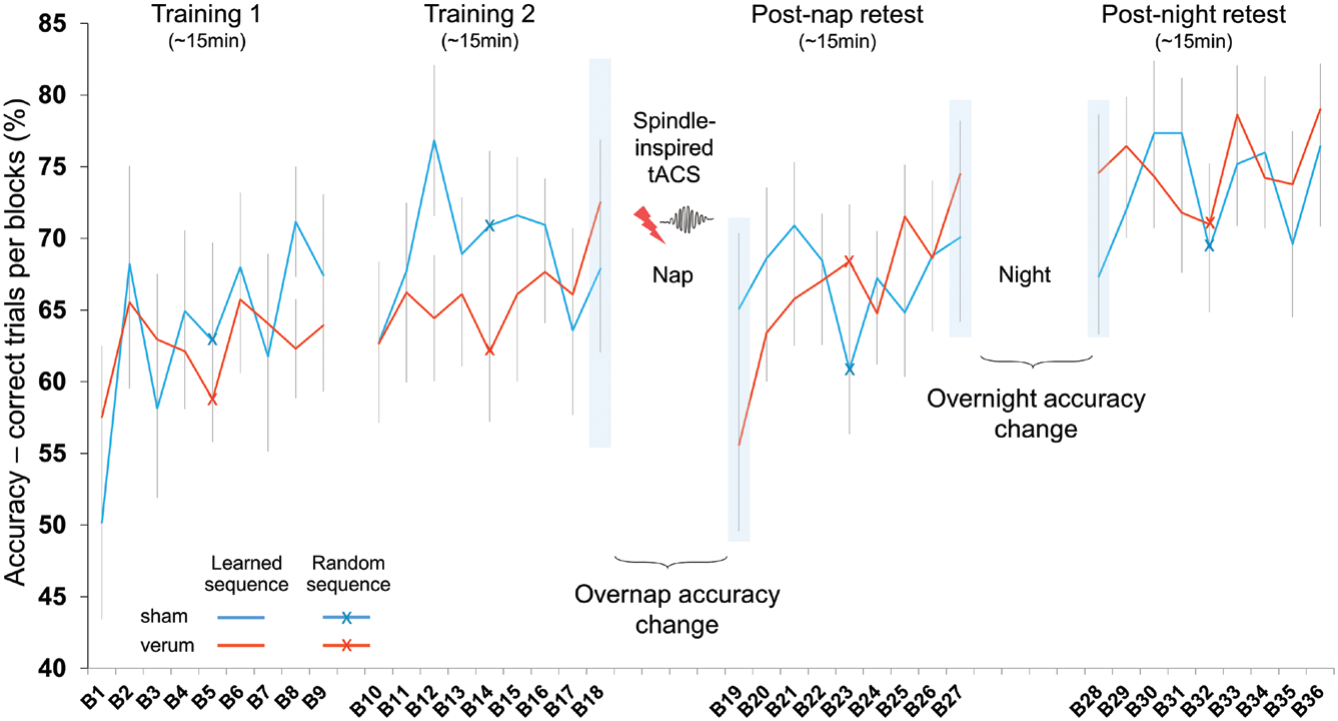
Overall motor learning dynamics in both groups. Learning curves of sham and verum groups. Each block depicts the average accuracy of 15 trials. Retest sessions were formed of 9 blocks, with the fifth block corresponding to the execution of a random sequence (cross). The morning training (training 1 and training 2) was followed by a nap during which participants received either the sham or the verum spindle-inspired tACS. The participants were then retested after the nap in the afternoon (post-nap retest) and after a night of sleep the next day (post-night retest). The shaded areas (at B18, B19, B27, and B28) depict the blocks used to calculate sleep-dependent motor memory consolidation (SDC): the overnap accuracy change and overnight accuracy change were calculated by the differences between pre-nap and post-nap blocks and pre-night and post-night blocks, respectively. Error bars represent the standard error of the mean (SEM).

Participants performed a sequential grip force modulation task that was adapted from Reis *et al.* [71] using MATLAB (MathWorks Inc., Sherborn, MA). The task was executed in the MRI scanner, and the MRI results are presented elsewhere [72]. Participants were trained during two sessions and subsequently retested after an afternoon nap and a night of sleep (Figure 1A). Sleepiness level was evaluated before each learning session with the Stanford Sleepiness Scale (SSS) [73]. Participants performed a total of three sessions of the sequential grip force modulation task: morning training and two retests (post-nap and post-night). During the training, participants performed two times nine blocks containing 15 targets (three repetitions of a sequence of five targets). Retests were shorter and contained nine blocks each (Figure 2). The fifth block of each training and retest corresponded to the execution of a random sequence that was different throughout the sessions (crossed line blocks in Figure 2) to test the learning rate of the sequence. The other blocks corresponded to the learned sequence that was the same throughout the experiment (smooth line blocks in Figure 2). The task involved applying force on a gripper that controlled the height of a cursor on a computer screen to match the height of a target bar. The force required to reach the target bars was adapted to the hand grip force of each participant, such that the upper bar corresponded to 70% of the individual maximum hand grip force. During the task, the participants were instructed to move the cursor to target bars in a sequence as fast and accurately as possible by pressing and releasing the gripper, and they were expected to learn to perform this sequence of trials, each of which demanded variable isometric force development of the non-dominant (left) hand (Figure 1B). A trial was correct when the cursor stayed for at least 200 ms between the upper and the lower limits of the aimed target. A trial was incorrect when the cursor stayed more than 200 ms on another target or between targets. The primary outcome was accuracy, which is evaluated by the proportion of trials that successfully reached target bars within a block (expressed in percentage, Figure 2). The speed to reach correct targets is computed per block as well as a compound measure combining accuracy and speed (accuracy in % divided by the average speed to reach targets) as secondary outcomes.

### Measurements of sleep-dependent motor memory consolidation

The primary goal of this study was to investigate whether spindle-inspired tACS enhanced sleep-dependent motor memory consolidation (SDC). The effect of sleep on SDC was defined as the difference in motor performance between the blocks flanking the sleep periods. The effect of the nap was computed as the difference between the post-nap block (B19) and the pre-nap block (B18), hereafter referred to as “overnap accuracy change.” The effect of the night was computed as the difference between the post-night block (B28) and the pre-night block (B27), hereafter referred to as “overnight accuracy change” (Figure 2).

### Spindle-inspired tACS and randomization

The current was delivered by a constant current stimulator (DC-Stimulator Plus, NeuroConn, Ilmenau, Germany) at 1 mA and replicated a spindle-inspired pattern sent by a Raspberry Pi through two concentric ring rubber electrodes. The small inner electrode had a diameter of 20 mm (3 cm^2^), and the external ring-shaped return cathode had a diameter of 100 mm (40 cm^2^). The spindle-inspired signal was created synthetically using custom scripts in Python and embedded in the Raspberry Pi. For safety reasons and keeping tES consensus guidelines, several digital and analogical circuits were put in place to ensure the robustness of the signal being delivered through the connection with the Neuroconn. The system was controlled with a custom graphical user interface that permitted the launch and interruption of the stimulation. The location of the electrode was guided by a hotspot determination with TMS (MagPro X100) (MagVenture, Farum, Denmark) using motor-evoked potentials recorded from the first dorsal interosseous muscle of the non-dominant left hand. We used an MC-B70 coil and monophasic pulses inducing a posterior to anterior current direction in the underlying brain tissue, with the handle pointing backward ~45°C relative to the midsagittal line. Electrodes were attached over the right motor cortex using an adhesive conductive gel (EC2 conductive paste) and held in place with a tubular-net.

**Figure 3.**
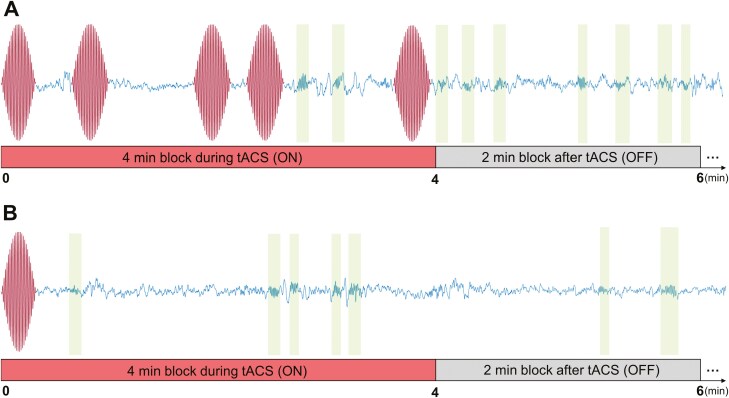
Schematic of spindle-inspired tACS. Spindle-inspired tACS was applied by blocks of 4 minutes ON and 2 minutes OFF during NREM2 and/or NREM3. The EEG trace from Pz electrodes shows artifacts when spindle-inspired tACS is applied (bursts on the trace). The spindles are highlighted in the shaded areas. Note that the time scale has been distorted for illustration purposes. (**A**) Spindle-inspired tACS in the verum group. Example of a 4-minute block ON followed by a 2-minute block OFF without stimulation. (**B**) Spindle-inspired tACS in the sham group, where each sham-stimulation block included one initial burst of tACS only.

tACS stimulations were presented in blocks of 4 minutes (Figure 3, ON blocks are represented in red), separated by stimulation-free intervals of 2 minutes (Figure 3, OFF blocks are represented in gray). The first stimulation block is started manually after 4 minutes of stable NREM sleep. Up to six stimulation blocks were applied, provided that the participant slept. The features of the bursts of tACS and their distribution are inspired by the spindle features that have been described in healthy young adults. These features are the duration (1.5 seconds [74, 75]), the frequency (14 Hz) corresponding to fast sigma range [76, 77], the waxing-waning shape (a ramp-in ramp-down of 0.5 seconds), and the density (44 spindles to obtain a spindle density of 11 bursts/min [36, 56, 75, 77–79]). The distribution of the bursts inside the ON blocks (Figure 3A, in red) was designed to reproduce the physiological inter-spindle intervals observed in young adults [80–83]. In the sham condition, the stimulation followed the ON/OFF block pattern except that only one burst of tACS was applied at the beginning of the ON block(s) (Figure 3B, in red). The stimulation was only performed during the nap.

Sleep was scored online using the standard guidelines [84], and the stimulation was stopped in case of awakenings or transitions back to NREM1, and the next block was launched after the next 1 minute of stable NREM2. Experimenters other than the online sleep scorer were blinded to the stimulation condition. After the nap, participants filled out a questionnaire (adapted from [85, 86]) about their sleep and sensations concerning the stimulation during the nap (see Supplementary Table S1). Participants were blinded to the stimulation condition throughout the study. After completing the entire study, they were asked which type of stimulation (sham or verum) was applied.

In the present experimental design, we expected that “natural” spindles would not appear frequently because the sleep duration of the nap was shorter than a night of sleep and, most importantly, because the number of spindles is known to be reduced in older adults compared with younger adults [47, 87, 88]. Therefore, we chose to apply tACS blocks independently of the natural ongoing oscillations to enable stimulation during a reasonable time during sleep.

### Polysomnography (PSG)

Electroencephalogram (EEG), electrooculogram (EOG), and electromyogram (EMG) were recorded continuously during the nap with Brain Vision Recorder using a VAmp Standard Amplifier (Brainproducts Inc., Gilching, Germany) at a 500 Hz sampling rate. During the nap, EEG was recorded from 12 scalp sites (F3, Fz, F4, C3, Cz, T3, T4, P3, Pz, P4, O1, and O2), according to the 10/20 system (Jasper 1958). To improve electric conductance, dead skin and sebum excess were eliminated from the scalp and face with NuPREPskin Prep Gel (Weaver and Company), and electrodes were attached to the scalp with EC2 Electrode Cream (NatusNeurology). Horizontal and vertical EOG were recorded with electrodes placed above the right outer canthus and below the left outer canthus, respectively. EMG was recorded with bipolar submental electrodes. Two electrodes placed on the mastoids were used as a common reference, and the ground was located on the left outer canthus. Impedances were kept below 5 kΩ.

### Data analysis

The overnap accuracy change was computed for 11 (sham) and 15 (verum) participants, respectively. Out of the 11 participants of the sham group, 10 continued the study on day 2. Thus, the overnight accuracy change (see below) of the sham group corresponds to 10 participants due to one missing data point.

#### Offline sleep scoring

Sleep scoring was conducted offline by two independent and experienced raters with the FASST toolbox run on Matlab R2016, following the criteria of the American Academy of Sleep Medicine [84, 89]. The interscorer agreement was above 90%. The continuous EEG was classified as wakefulness (W), NREM1, NREM2, NREM3, or rapid eye movement (REM) sleep using 20-second epochs. Epochs with large blinks or movement artifacts were removed with FASST from further analysis as a prior step before artifact rejection during signal preprocessing that was performed using Fieldtrip [90]. Arousals were also identified with FASST [84].

Two types of analysis were performed to estimate sleep features during the nap. The first analysis included all the epochs to estimate the real sleep duration in both groups (Table 1, entire nap). The second sleep analysis excluded stimulated epochs from the sleep calculation. Indeed, the large artifacts of spindle-inspired tACS prevented sleep staging for the 20-second epochs with 100% of confidence in the verum group (Figure 3). To compare this group with the sham group, markers were placed in the sham recordings to indicate when blocks of stimulation would have ended, and these segments were also excluded from the analysis (Table 1, stimulation epochs excluded).

**Table 1.** Sleep architecture of the nap in the sham and verum groups

	Sham Mean ± SD	Verum Mean ± SD	Statistics *F*-value
**Entire nap**			
TIB (min)	73.2 ± 13.9	70.5 ± 10.4	0.32
TST (min)	51.6 ± 14.2	57.7 ± 8.9	1.84
Sleep efficiency (%)	70.5 ± 15.3	81.8 ± 12.6	2.49
NREM (2 and 3; min)	38.3 ± 12.5	42.6 ± 11.6	0.81
NREM1 onset latency (min)	4.9 ± 3.2	2.9 ± 1.8	4.02
NREM2 onset latency (min)	8.2 ± 4.0	5.8 ± 3.5	2.65
Wake after sleep onset (min)	16.7 ± 12.3	9.8 ± 10.0	2.48
Arousal number	22.9 ± 17.8	14.9 ± 9.9	2.13
Mean arousal duration (sec)	7.9 ± 1.8	8.8 ± 2.8	0.76
Arousal time (min)	1.9 ± 1.2	2.1 ± 1.3	0.08
**Stimulation epochs excluded**			
Wake (min)	19.8 ± 11.5	11.9 ± 11.3	2.97
NREM1 (min)	10.8 ± 7.3	12.4 ± 8.8	0.24
NREM2 (min)	17.9 ± 6.9	20.4 ± 5.9	0.93
NREM3 (min)	4.2 ± 4.2	6.1 ± 7.3	0.59
REM (min)	0.0 ± 0.0	1.0 ± 1.8	3.52
TST (min)	32.9 ± 9.9	39.9 ± 7.7	4.09
NREM1 (%)	18.4 ± 9.8	21.6 ± 12.13	0.50
NREM2 (%)	33.6 ± 12.6	39.4 ± 11.8	1.47
NREM3 (%)	9.2 ± 11.1	13.0 ± 15.8	0.46
NREM1/NREM ratio	30.7 ± 14.8	30.5 ± 18.7	0.001
NREM2/NREM ratio	55.5 ± 16.4	51.7 ± 13.1	0.43
NREM3/NREM ratio	13.9 ± 16.2	15.2 ± 16.7	0.04

SD, standard deviation; min, minutes; %, percentage; TIB, time in bed; TST, total sleep time; REM, rapid eye movement sleep; NREM, non-rapid eye movement sleep.

TST is the sum of NREM and REM sleep; NREM is the sum of NREM1, NREM2, and NREM3 sleep stages; sleep efficiency is the percentage of sleep relative to TIB and REM. Group *F*-tests were performed with *sumtable* function.

#### Spindle detection and calculations

Spindles were detected visually on the Pz channel (during the entire nap) by two independent and experienced raters using Fieldtrip on Matlab R2016 and RemLogic v4.0.1 (Embla Systems, Natus Medical Incorporated, Canada). A duration threshold enabled to eliminate the spindle lasting less than 200 ms [40]. The number and density of spindles per minute of NREM sleep (sp/min) were computed. A second measure of spindle density including the tACS bursts was also calculated as corresponding to the number of physiological spindles plus the number of tACS bursts per minute of sleep. If not mentioned otherwise, spindles are the naturally occurring physiological spindles observed on the EEG trace during the nap.

#### Statistics

Statistical analyses were conducted using RStudio (RStudio Core Team, 2020). To evaluate the effects of spindle-inspired tACS on overnap accuracy change and overnight accuracy change, we built a linear mixed model using the *lmer* function in the *lme4* package. As fixed effects, we added Time as a within-subject factor (two levels: pre- and post-sleep) and Group as between-subject factor (two levels: sham and verum). We then investigated the effects of spindle-inspired tACS on sequence learning by computing performance on the trained sequences (mean accuracy of the blocks flanking the random sequence of each session, i.e. fourth and sixth blocks) and on the random sequence (mean accuracy on the fifth block of each session). We then entered these values in a linear mixed model, with Sequence Type as within-subject factor (two levels: random, learned), Session as within-subject factor (four levels: training 1, training 2, post-nap retest, post-night retest), and Group as between-subject factor (two levels: sham, verum). For linear mixed models, the factor subject was taken as a random intercept, and statistical significance was determined using the *anova* function in the *lmerTest* package. For the main results with linear mixed models, partial eta-squared (η^2^_p_) values are presented as measures of the standardized effect sizes where applicable (*eta_squared* function in the *effectsize* package).

As previous studies have shown associations between spindles and memory consolidation [13, 36, 52], we estimated linear models (function *lm* in R) including the factor Group (two levels: sham, verum), Spindle density, and Group × Spindle density interaction terms. The dependent variables were overnap accuracy change and overnight accuracy change; they were modeled separately. As a further supportive analysis, the contribution of the bursts of tACS for overnap accuracy change and overnight accuracy change was also tested using separated linear models. Linear models were also used to estimate the effects of the stimulation on spindle density, sleep architecture, and demographic features using the *sumtable* function (ANOVAs). For all statistical analyses, a two-sided significance level of α = 0.05 was considered.

## Results

### Participant characterization

There were no significant differences between sham and verum participants in terms of age, years of formal education, general cognitive status (MoCA), physical activity (hours of exercise per week), sleep quality (PSQI), attentional level during the motor task sessions (SSS), and motor assessments (grip force test), but there was a greater proportion of females in the sham group compared to the verum group, which was reflected in a higher right-hand maximal fist grip force in the verum group (*p* = 0.0065) but not in the left hand (see Supplementary Table S3). Across both groups, no significant differences were observed in sleep parameters as a function of gender and grip force. The sham control was effective; participants were unable to guess beyond chance level which type of stimulation was administered (Supplementary Table S1 for tACS-related reported sensations).

### Sleep parameters and spindle-inspired tACS during the nap

The 26 participants slept for 24.7 ± 7.3 minutes (range: 12–40 minutes of NREM2 and NREM3) during the 71.6 ± 11.6 minutes opportunity to nap (time in bed). Note that out of these 26 participants, one did not continue the experiment on day 2, hence a lower number of participants to estimate the overnight consolidation (*N* = 10 in the sham group). The sham and verum groups did not differ on any sleep parameters (see Table 2 for details). Data of participants who did not sleep (i.e. the no-nap group) are described in the supplementary information (Supplementary Figure S1).

**Table 2.** Linear model results for overnap (A) and overnight (B) accuracy changes

(A) Overnap accuracy change ~ Group × Spindle density				
Predictors	Estimates	CI		*p*
(intercept)	−6.73	−37.48	24.04	0.655
Group	−34.45	−78.18	9.49	0.118
Spindle density	1.27	−7.45	10.00	0.765
Group × Spindle density	7.04	−6.13	20.21	0.280
observations	26			

(B) Overnight accuracy change ~ Group × Spindle density				
Predictors	Estimates	CI		*p*
(intercept)	−31.33	−52.31	−10.36	0.00534
Group	46.81	17.27	76.35	0.00345
Spindle density	8.34	2.50	14.17	0.00727
Group × Spindle density	−13.63	−22.41	−4.84	0.00406
observations	25			

Linear model analyses were conducted with the sham group as the reference category. For the overnap model, the interaction was excluded from the model to see the main effect of Spindle density since the interaction was not significant (*p* = 0.182).

#### Spindle-inspired tACS during the nap

Spindle-inspired tACS has been applied according to each participant’s ability to sustain stable NREM sleep (see above). Hence, the number of blocks varied between the participants: they received between one and six blocks of stimulation (sham group: 5.0 ± 1.5; verum group: 4.4 ± 1.8), for a total stimulation duration of 17.2 ± 6.3 minutes representing 25.1 ± 10% of the nap (Supplementary Table S2). As we injected artificial spindles (i.e. bursts of tACS) in the verum group, the sum of artificial and naturally occurring physiological spindles was three times higher in the verum group compared with the sham group, resulting in a higher spindle density including the tACS bursts in the verum group (Supplementary Figure S2).

#### Spindle-inspired tACS effects on sleep and spindles during the nap

We evaluated whether spindle-inspired tACS changed sleep macrostructure based on the visually scored sleep stages (Table 1). Spindle-inspired tACS did not influence the total sleep time and the duration of NREM2 and NREM3. Spindle-inspired tACS did not influence sleep by inducing more arousal. However, the sleep efficiency was slightly higher in the verum group compared with the sham group, as the results of a lower wake duration and a higher NREM duration in favor of the verum group when the stimulation epochs were excluded from the analysis (Table 1). We also assessed whether spindle-inspired tACS modulated the number, density, and duration of the naturally occurring physiological spindles. A similar number of spindles was produced during the nap in the sham and verum groups (Supplementary Figure S3A). Similarly, spindle density (relative to the NREM sleep duration) and spindle duration did not differ significantly between the sham and the verum group (Supplementary Figure S3B and S3C).

### Learning of the sequential grip force modulation task

To verify that sequence learning occurred in both groups, we examined the accuracy of the learned and random sequences over the training and the retest sessions. For each session, the learned sequence was computed as the mean of the blocks flanking the random sequence (the fourth and the sixth blocks), and the random sequence was the fifth block (Figure 2). The accuracy improved over the sessions as shown by a significant main effect of Session (training 1, training 2, post-nap retest, and post-night retest) (*F*_(3, 166)_ = 5.65, *p* < 0.001, η^2^_p_ = 0.09). We observed no main effect of Group (sham, verum) (*F*_(1, 24)_ = 0.18, *p* = 0.678, η^2^_p_ = 0.01), while there was a main effect of Sequence type (learned, random sequence) (*F*_(1, 166)_ = 3.92, *p* = 0.0494, η^2^_p_ = 0.02). We observed an absence of a triple interaction Group × Sequence type × Session (*F*_(3, 166)_ = 0.77, *p* = 0.511, η^2^_p_ = 0.01) and of double interactions: Group × Session (*F*_(3, 166)_ = 1.34, *p* = 0.264, η^2^_p_ = 0.02), Session × Sequence type (*F*_(3, 166)_ = 0.26, *p* = 0.851, η^2^_p_ = 0.00), and Group × Sequence type (*F*_(1, 166)_ = 0.18, *p* = 0.672, η^2^_p_ = 0.00). Taken together, this pattern of results suggests that the trained sequence was learned to a larger extent compared with the random sequences over the sessions but was not influenced by the spindle-inspired tACS.

### Effects of spindle-inspired tACS on changes of overnap and overnight consolidation

Before looking at the effects of spindle-inspired tACS, we ensured that the motor task was learned similarly in both groups ahead of the application of the stimulation and the group effect was absent.

#### Overnap consolidation

**Figure 4.**
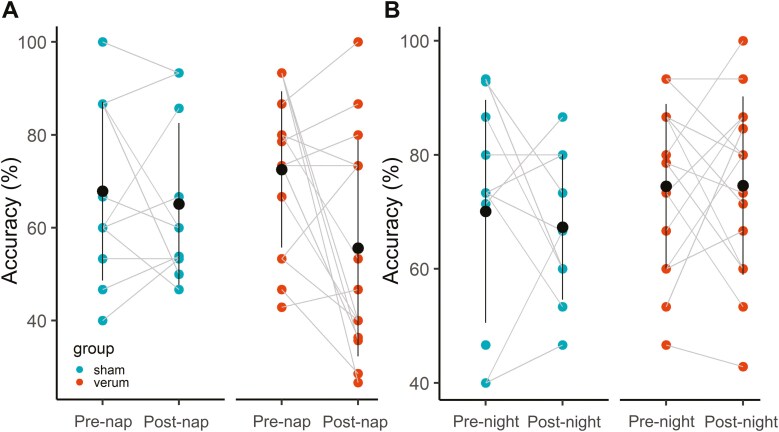
Effects of spindle-inspired tACS on overnap and overnight accuracy change. (**A**) Overnap changes in pre-nap and post-nap performance accuracy. (**B**) Overnight changes in pre-night and post-night performance accuracy. The sham group (left) (pre-post nap: *N* = 11, pre-post night: *N* = 10) and the verum group (right) (*N* = 15) are depicted, respectively. The circles with the error bars (vertical lines: SD) indicate the mean values. Spindle-inspired tACS did not significantly modulate overnap accuracy change and overnight accuracy change.

To test for differential overnap accuracy change more specifically in the sham and verum groups, we compared the block before the nap (pre-nap accuracy) to the block after the nap (post-nap accuracy: Figure 4A). The effect of Time was significant (*F*_(1, 24)_ = 5.51, *p* = 0.0274, η^2^_p_ = 0.15), but the effect of Group was not significant (*F*_(1, 24)_ = 0.15, *p* = 0.697, η^2^_p_ = 0.01). Additionally, the interaction term Time × Group was also not significant (*F*_(1, 24)_ = 2.26, *p* = 0.146, η^2^_p_ = 0.09). Napping led to a decrease in motor performance equally in both groups; spindle-inspired tACS did not show a significant behavioral effect compared to sham stimulation.

#### Overnight consolidation

We also tested for any differential effects of tACS on subsequent overnight consolidation processes. To this end, we compared accuracy measures for the last learned block before the night (pre-night accuracy; block 27) to the first learned block after the night (post-night accuracy; block B28) and tested if overnight accuracy change differed between groups (Figure 4B). The effect of Time and the effect of Group were not significant (*F*_(1, 23)_ = 0.20, *p* = 0.660, η^2^_p_ = 0.01; *F*_(1, 233)_ = 1.47, *p* = 0.238, η^2^_p_ = 0.06, respectively). Additionally, the interaction Time × Group was also not significant (*F*_(1, 23)_ = 0.23, *p* = 0.638, η^2^_p_ = 0.01). These results suggest that sleeping during the night led to motor performance stabilization equally in both groups, i.e. no effect of the presence of spindle-inspired tACS during the preceding nap.

Beyond the null effect of spindle-inspired tACS on overnap and overnight consolidation, these analyses indicated that daytime sleep and nighttime sleep may have different effects on the learning of the grip task. Indeed, we observed a decrease in motor performance from pre- to post-nap (significant main effect of time for overnap accuracy change) while a night stabilized motor performance (absence of overnight accuracy loss). Moreover, sleep duration during the nap was not associated with overnap accuracy changes, suggesting that the nap-related decrease of accuracy was not explained by the sleep duration during the nap. Finally, additional analyses on a group of participants who remained awake during the nap time showed that offline motor performance was not impacted by the presence of a nap compared with no-nap (see Supplementary Figure S1).

### Effects of spindle density on behavioral outcomes

The secondary analyses examined the associations between the natural spindle density and overnap and overnight accuracy changes, and whether the stimulation modulated these associations.

#### Effect of spindles on overnap consolidation

**Figure 5.**
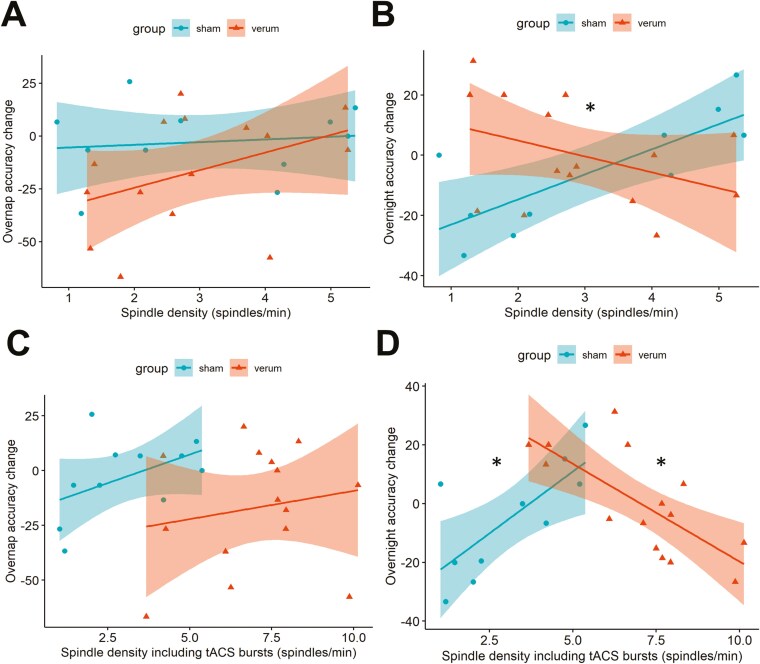
Table 2Correlations between spindle density during the nap and overnap or overnight accuracy change. (**A**) Overnap accuracy change and spindle density. (**B**) Overnight accuracy change and spindle density during the nap. (**C**) Overnap accuracy change and spindle density, including tACS bursts (number of spindles and tACS bursts during the nap). (**D**) Overnight accuracy change and spindle density, including tACS bursts (number of spindles and tACS bursts during the nap). The sham group is depicted with the circles (overnap: *N* = 11; overnight: *N* = 10); the verum group (*N* = 15, for both overnap and overnight) is depicted with the triangles. The shaded areas depict 95% confidence intervals. Statistical analyses were conducted using linear models including the factors Group, Spindle density, Group × Spindle density interaction term (Table 2). Statistical significance markers: *p < 0.05.

Linear model analysis did not reveal a significant main effect of Group (*p* = 0.118), Spindle density (*p* = 0.765), and Group × Spindle density interaction (*p* = 0.278) (Table 2A and Figure 5A).

#### Effects of spindles on overnight consolidation

Results of linear model analysis showed a significant Group × Spindle density interaction (*p* = 0.004) (Table 2A and Figure 5B), indicating that spindle density associates differently with overnight accuracy change according to the group (Table 2B): a positive association between spindle density and overnight accuracy change was reported in the sham group (*p* = 0.012) but not in the verum group (*p* = 0.490).

Similar analyses performed with speed of the task yielded comparable results (see Supplementary Figure S6), except that the spindle density was not associated with overnight speed change in the sham group (see Supplementary Figure S7). The results of the compound measure point toward a similar direction as accuracy results (data not shown).

### Effects of spindle density, including tACS bursts, on behavioral outcomes

#### Overnap consolidation

The correlations between spindle density, including tACS bursts, and overnap accuracy change were not significant in the sham (*r* = 0.47, *p* = 0.14) and the verum group (*r* = 0.18, *p* = 0.52) showing that the spindle density combined with tACS bursts did not influence overnap accuracy change (Figure 5C).

#### Overnight consolidation

While overnight accuracy change correlated positively with spindle density, including tACS bursts, in the sham group (*r* = 0.74, *p* = 0.015), the correlation was negative in the verum group (*r* = −0.72, *p* = 0.003; Figure 5D). These results converge with the previous analyses performed on physiological spindle density, thus further emphasizing the hampering effects of spindle-inspired tACS on overnight accuracy change when the spindle density is inflated (by the adjunction of tACS bursts).

To summarize these results, spindle density during nap did not show a correlation with overnap accuracy change (Figure 5A), but with the subsequent overnight accuracy change in the sham group, but not in the verum group (Figure 5B). Specifically, participants in the sham group who presented higher spindle density had higher overnight accuracy change, confirming that natural spindles support consolidation, which might be primarily driven by NREM2 spindles (see Supplementary Figure S4). The same correlation in the sham group was found when spindle density included tACS bursts. Intriguingly, when including tACS bursts, the verum group displayed an inverted correlation, whereby participants with lower overall spindle density showed better overnight consolidation (Figure 5D). This pattern of results thus suggests that spindle-inspired tACS during a nap may disrupt subsequent overnight consolidation.

## Discussion

This study aimed to investigate the effects of spindle-inspired tACS during a nap on overnap and overnight memory consolidation in healthy older adults. We hypothesized that spindle-inspired tACS applied in this population during a short daytime sleep period (i.e. a nap) would enhance spindle activity and boost SDC. The present results demonstrated an association between spindles and SDC in healthy older adults (Figure 5). However, contrary to our initial expectation, spindle-inspired tACS did not enhance the density of the natural spindles or memory consolidation (Figure 4). We discuss below possible reasons for these null effects, and how they may inform future tES investigations aimed at modulating sleep features to boost memory consolidation.

Sleep spindles play a strong functional role in memory consolidation [30, 52, 91], and can be modulated non-invasively [64]. Spindle-inspired tACS, mimicking naturally occurring spindles, thus appears as a very promising tool to enhance motor memory consolidation. This might be particularly relevant for older populations in whom the reduction of sleep spindles might contribute to deficits in SDC [30, 43, 50]. To enhance feasibility, if successful, we focused on short sleep bout (nap) rather than nighttime sleep. Indeed, targeting naps has the following advantages. Daytime napping behavior is commonly reported in older adults [92–98]; naps reveal comparable memory benefits as night sleep [10, 12, 13] and exhibit similar oscillatory features (i.e. spindle density [99]). tES has previously been shown to be effective in improving memory when applied during night and nap [55, 61, 100, 101]. Additionally, from a practical feasibility standpoint, naps avoid the more difficult setup of nocturnal stimulation protocols (i.e. stress induced by late-night testing) and thus enhance translational relevance. Here, we aimed at increasing the number of sleep spindles to a level that is physiologically described in young adults by the application of tACS bursts. Artificial spindles were applied independently of the occurrence of natural sleep spindles, which are known to be much reduced in this cohort [47, 49, 87]. Despite the convincing theoretical background, the present empirical data did not support the hypothesis that spindle-inspired tACS enhanced memory consolidation in healthy older adults. We found an absence of significant difference in the consolidation of a motor task in the verum and sham groups (Figure 4).

Although tACS targeting the spindles has never been tested in older adults, our results can be compared with the studies that targeted the SW with so-tDCS to enhance memory in older adults. These studies showed heterogenous effects on sleep physiology and memory (for reviews, see [62, 63]), yet no significant effect has been found on motor memory consolidation. From the results of the seminal study by Marshall *et al.* [56, 58], where only declarative memory was enhanced, motor tasks were rather used as control tasks in the next published studies. Our results are in line with these reports showing an absence of stimulation effects on motor memory consolidation when applied during a night [59, 60] or a nap [55, 61]. The study by Ladenbauer *et al*. tested the effects of so-tDCS on different memory tasks at the same time. They found that performance on a sequential finger-tapping task improved after a nap, but that so-tDCS did not further enhance motor performance, whereas explicit visual memory recognition was enhanced. Besides, so-tDCS boosted the slow-wave and spindle activities. Westerberg *et al*. also found that so-tDCS enhanced word-pair recall and slow-wave activity, yet they did not test the effects of so-tDCS on procedural memory. The similarity between these studies and our protocol is that the stimulation was applied independently from the ongoing sleep oscillations (open-loop manner), raising the question of whether, for procedure memory consolidation processes, stimulations should be time-locked to the oscillations of interest [59, 60, 102, 103]. Along this line, spindle-inspired tACS time-locked to the natural spindles has been found to enhance motor memory consolidation through spindle activity enhancement in young adults [64]. tACS is assumed to operate via the entrainment of endogenous brain oscillations of matching tACS frequency [104, 105]. Here, the application of the spindle-inspired tACS occurred independently from the natural spindle occurrence, so the bursts might have occurred inside and outside of the spindles. We reported non-significant changes in natural spindle density during the nap between the sham and the verum groups, suggesting that even though the stimulation was tuned toward a physiological spindle pattern and designed according to physiological spindle features, the spindle generation was not modulated. One possible explanation for this is that open-loop stimulation might have rather disrupted the temporal organization of spindles (see Supplementary Figure S5), which is relevant for memory [22, 31, 106], and might be reflected in the negative relationship with overnight accuracy change we observed (Figure 5D). Taken together, these findings indicate that open-loop stimulation might not be an efficient strategy to enhance the generation of sleep spindles, and that closed-loop stimulation approaches might be mandatory to achieve behavioral and/or sleep-physiological effects.

Other factors might explain the absence of tACS effects in the present study. Indeed, the motor cortex might not have been the perfect target region to enhance motor memory consolidation since several brain regions, upstream of the primary motor cortices, are critically involved in motor learning [72, 107]. Other deep brain structures, such as the hippocampus or the thalamus [2, 108], are critical for sleep oscillations and memory consolidation and may be considered as targets for an interventional approach [109–111]. So far, these structures could not be reached with non-invasive brain stimulation (NIBS) methods. However, recent findings in rodent work provided evidence for a novel opportunity to apply physiology-inspired electrical stimulation to deep structures with good focality [112], recently validated in humans showing a good safety and efficiency profile [113–116]. These findings are very promising and open a new opportunity to target sleep- and consolidation-relevant deep brain structures non-invasively.

The natural spindle density during the nap in the sham group did not correlate with motor memory consolidation post-nap (Figure 5A), which was also shown in a recent study [117]. While Fogel *et al.* suggested that the relationship between motor skills and spindles deteriorates with age [40], a recent nap study found that this relationship is preserved: a poor memory consolidation of motor skill is reflected by a slight spindle increase, which seems insufficient for a proper memory consolidation [50]. In the present study, the absence of an association with the immediate nap consolidation and the presence of an association with the night consolidation suggest that memory of the motor skill was physiologically tagged during the nap through the spindles [118–120] and was subsequently consolidated during the sleep night, a process that plausibly benefited from the succession of NREM–REM cycles [121]. The results of this study point toward different mechanisms during day and night sleep and thus encourage the investigation of the stimulation also during the night. They also raise the question of the potential influence of sleep oscillations during a nap on the following night of sleep and memory consolidation.

The lack of significant results raises the question of sample size. The final sample resulted from a highly selective inclusion process and from a highly demanding experimental design, involving multiple visits covering behavioral tests inside and outside the scanner, and requiring the participants to sleep on demand during the day. Here, we analyzed data from a final sample of 26 older participants, which was the result of high drop-out and reallocation rates. The COVID pandemic significantly impacted the progress of the study during its duration; thus, although the sample size is small, we concluded that the sample size achieved was near adequate to yield meaningful outcomes for the tested factors considering the difficulties in the study. Critically, adding more participants would probably not change our interpretation of a null or deleterious effect of the spindle-inspired tACS, since the verum group did not demonstrate any trend toward better overnap or overnight performance.

## Conclusion

The present study used spindle-inspired tACS to create a high density of sleep spindles during a nap and enhance SDC in an older population. The present data confirm the relevant role of sleep spindles and their potential as an interventional target for NIBS to boost motor memory consolidation in healthy older adults. Contrary to spindle-inspired tACS time-locked to ongoing spindles during night sleep and targeting a broad network [64], the present non-state-dependent protocol, focused on the motor cortex, did not enhance memory consolidation. Enhancement of oscillatory sleep activity using tACS to foster memory consolidation processes is promising; however, the present results suggest that it might be critical that tACS is applied in tight temporal association with the ongoing sleep oscillations, i.e. in a closed-loop state-dependent manner, and favored during a night sleep over short nap designs given the potential scarcity of relevant sleep oscillations in older populations.

## Supplementary Material

zpaf022_suppl_Supplementary_Materials

## Data Availability

The data underlying this article will be shared on reasonable request to the corresponding author.
