# Supplementary material for: Impact of spindle-inspired transcranial alternating current stimulation during a nap on sleep-dependent motor memory consolidation in healthy older adults
Source: Sleep Adv. 2025 Mar 26;6(2):zpaf022. doi: 10.1093/sleepadvances/zpaf022 (PMC12070486; doi:10.1093/sleepadvances/zpaf022)
Supplement: zpaf022_suppl_Supplementary_Materials [file zpaf022_suppl_supplementary_materials.docx]

Supplementary information for

**Impact of spindle-inspired transcranial alternating current stimulation during a nap on sleep-dependent motor memory consolidation in healthy older adults**

Moyne M.^1,2,3^, Durand-Ruel M.^1,2^, Park C.^1,2^, Salamanca-Giron R.^1,2^, Sterpenich V.^4,5^, Schwartz S.^5^, Hummel F.C.^1,2,3^ & Morishita T.^1,2^

^1^ Defitech Chair of Clinical Neuroengineering, Neuro X Institute (INX), École Polytechnique Fédérale de Lausanne (EPFL), Geneva, Switzerland.

^2^ Defitech Chair of Clinical Neuroengineering, INX, EPFL Valais, Clinique Romande de Réadaptation, Sion, Switzerland.

^3^ Clinical Neuroscience, University of Geneva Medical School, Geneva, Switzerland.

^4^ Fondation Campus Biotech Geneva, Geneva, Switzerland.

^5^ Department of Basic Neurosciences, University of Geneva Medical School, Geneva, Switzerland.

**Correspondence:**

Dr. Takuya Morishita

Defitech Chair of Clinical Neuroengineering, Neuro X Institute (INX), École Polytechnique Fédérale de Lausanne (EPFL), Campus Biotech, Chemin des Mines 9, 1202 Geneva, Switzerland.

Email: [takuya.morishita@epfl.ch](mailto:takuya.morishita@epfl.ch)

# **No-nap group: effects of napping on Overnap accuracy change and Overnight accuracy change**

The success of falling asleep during the day is not 100%, mostly in the laboratory environment. Knowing this probability, all the participants were invited to sleep after lunch. If they were unable to sleep after an hour of attempts, they were reallocated to the no-nap group. Participants listened to an emotionally neutral podcast to keep the participants awake with their eyes closed while three blocks of spindle-inspired tACS were applied under EEG monitoring to make sure that they stayed awake. Five participants initially planned in the nap group were reallocated to the no-nap group because they failed to fall asleep. Five more participants were included in the no-nap group without prior daytime sleep attempts. A total of ten healthy older adults (6 females, mean ± SD, 67.8 ± 5.26 years) acted as wake controls to estimate SDC.

SDC is a complex process that is not fully understood yet. Since many years, literature demonstrated the importance of sleep in motor learning process in young adults (Walker & Stickgold, 2004, but see Schmid *et al.*, 2020). Many reports found an absence of significant motor performance change after a wake interval (wake stabilizes motor skill) while after sleep, motor performance improved consequentially (Walker *et al.*, 2002; Walker, 2005; Nishida & Walker, 2007; Doyon *et al.*, 2009; Debarnot *et al.*, 2011; Schmid *et al.*, 2020). However, with age SDC reduction is generally reported such that sleep does not lead to additional motor performance gains relative to wake (King *et al.*, 2017; Gui *et al.*, 2017). Moreover, nap might stabilize motor performance by exhibiting a delayed benefit visible after a nocturnal sleep period (Korman *et al.*, 2015). Therefore, SDC of the nap group is compared with the no-nap group to assess the immediate (Overnap accuracy change) and delayed (Overnight accuracy change) effects of napping on SDC.

To evaluate the effects of napping on Overnap accuracy change and Overnight accuracy change a linear mixed model was performed using the *lmer* function in the *lme4* package. As fixed effects, time as within-subject factor (2 levels: pre, post-sleep) and group as between-subject factor (2 levels: nap, no-nap) were added.

## Immediate effect of napping on Overnap accuracy change

We compared the block before the intervention (pre-break accuracy) to the block after the intervention (post-break accuracy) and tested if Overnap accuracy change differed between groups. The effect of Time was not significant (F_(1,21)_ = 1.384, p = 0.252) as well as the effect of Group (F_(1,21)_ = 0.394, p = 0.537. Additionally, the interaction term Time × Group was also not significant (F_(1,21)_ = 0.133, p = 0.718, Fig. S1A), therefore the accuracy stabilized in both groups suggesting that napping did not lead to additional motor gains compared with staying awake during the afternoon break.

## Delayed effect of napping on overnight accuracy change

We compared the block before the night (pre-night accuracy) to the block after the night (post-night accuracy) and tested if Overnight accuracy change differed between the groups. The effect of Time was not significant (F_(1,19.2)_ = 1.751, p = 0.201) as well as the effect of Group (F_(1,19,7)_ = 1.480, p = 0.238). Additionally, the interaction term Time × Group was also not significant (F_(1,19.2)_ = 0.199, p = 0.660, Fig. S1B), therefore the accuracy stabilized in both groups suggesting that napping did not bring additional overnight motor gains compared with staying awake during the afternoon break.

Studies including older adults often aimed at comparing them to a younger group to evaluate the age-related change in SDC. The age comparison is out of the scope of this study because the evaluation of the motor learning in a young cohort has not been planned in the study design. However, we can compare our results to the older adult group of the former studies.

Our results show that napping did not lead to additional motor gains right after the afternoon break and after the subsequent night of sleep. Sleep-independent motor performance stabilization has been previous found in older adults (Backhaus et al., 2016; Gui et al., 2017; Mander et al., 2017) after a nap (Fogel *et al.*, 2014; Backhaus *et al.*, 2016*a*; King *et al.*, 2016; Vien *et al.*, 2016; Fang *et al.*, 2021). Such findings are consistent with previous studies demonstrating that an afternoon nap did not enhance motor sequence performance in older adults (Fogel *et al.*, 2014; Korman *et al.*, 2015; Backhaus *et al.*, 2016*b*). Our results contrast reports showing night-related additional motor performance increase (King *et al.*, 2016; Mander *et al.*, 2017*b*) or decrease (Backhaus *et al.*, 2016*a*). Then, our results indicate that napping in the afternoon does not impact the subsequent overnight stabilization (Backhaus *et al.*, 2016*b*). Our results are contrasting the few reports showing larger sleep-related improvement in older adults (Tucker *et al.*, 2011; Gudberg *et al.*, 2015; King *et al.*, 2016), however these motor improvement are observed after extra practice or the result of less dexterous practice involving larger body parts (Gudberg *et al.*, 2015).

Altogether, our results support reports demonstrating that sleep stabilizes motor performance in older adults after a nap (Vien *et al.*, 2016; Fang *et al.*, 2021) and a night of sleep (Backhaus *et al.*, 2016*c*; Gui *et al.*, 2017). These results are also in line with young adults’ studies stating that sleep does not enhance but rather stabilized memory (Robertson *et al.*, 2004; Fogel *et al.*, 2014; Maier *et al.*, 2017) for reviews see (Pan & Rickard, 2015; Schmid *et al.*, 2020).


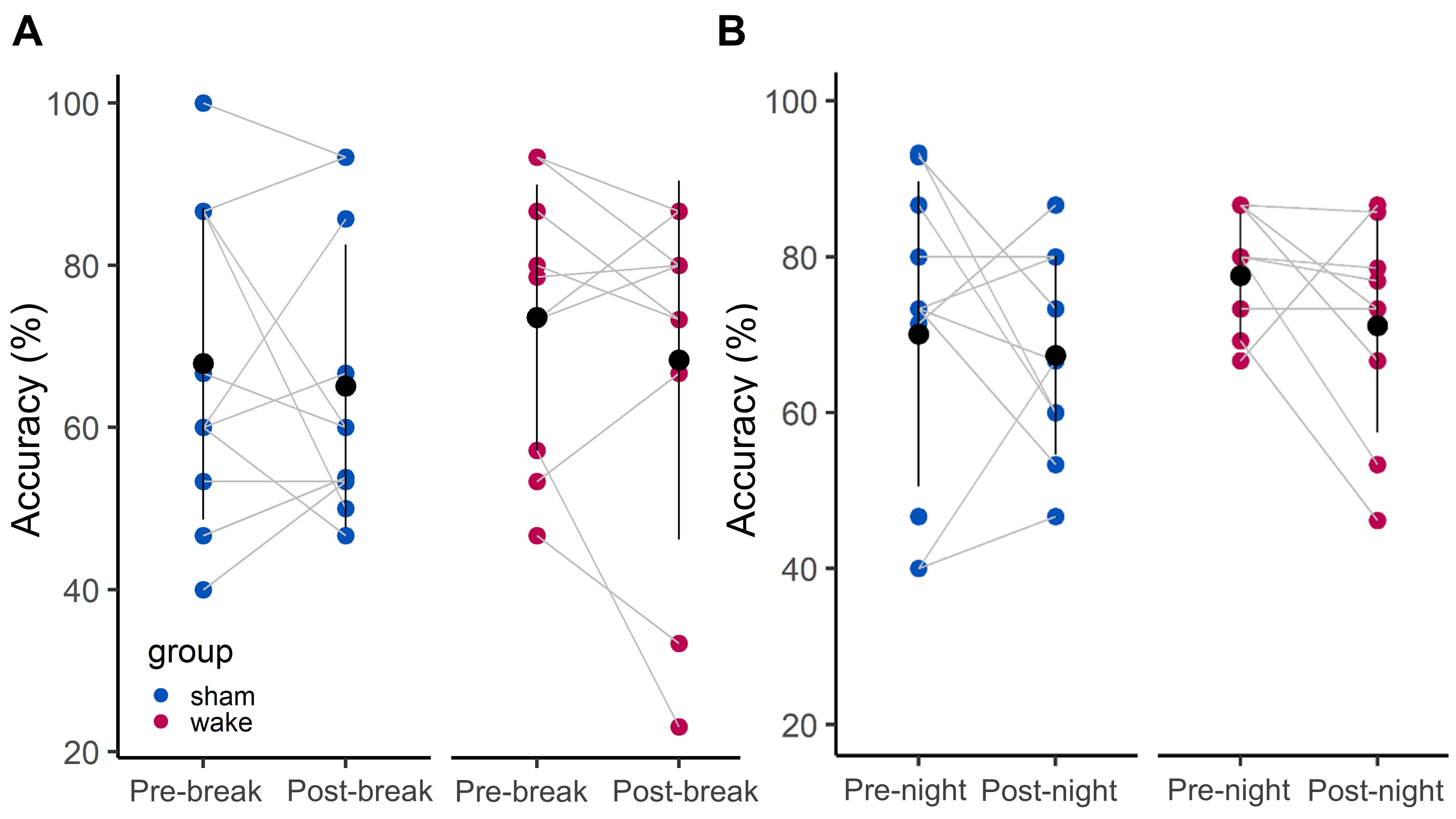


**Figure S1. Effect of napping on Overnap accuracy change and Overnight accuracy change.** (**A**) Overnap accuracy change. (**B**) Overnight accuracy change. The dark blue circles depict the sham group (nap group) (overnap: N = 11; overnight: N = 10) and the dark red circles depict the no-nap group (N = 10). The block circles indicate the mean values, and the vertical lines are the standard deviation. Overnap accuracy change and Overnight accuracy changes were similar in both groups. Napping did not modulate memory consolidation.

# **Sleep spindle data**

In the following sections, we demonstrate details of sleep spindle data presented in the study as well as further analyses.

## Number of spindles and tACS bursts

Fig. S2 shows the number of spindles and tACS bursts during the nap. The spindle density including tACS bursts was larger in the verum group compared with the sham group as expected (Fig. S2B).

**
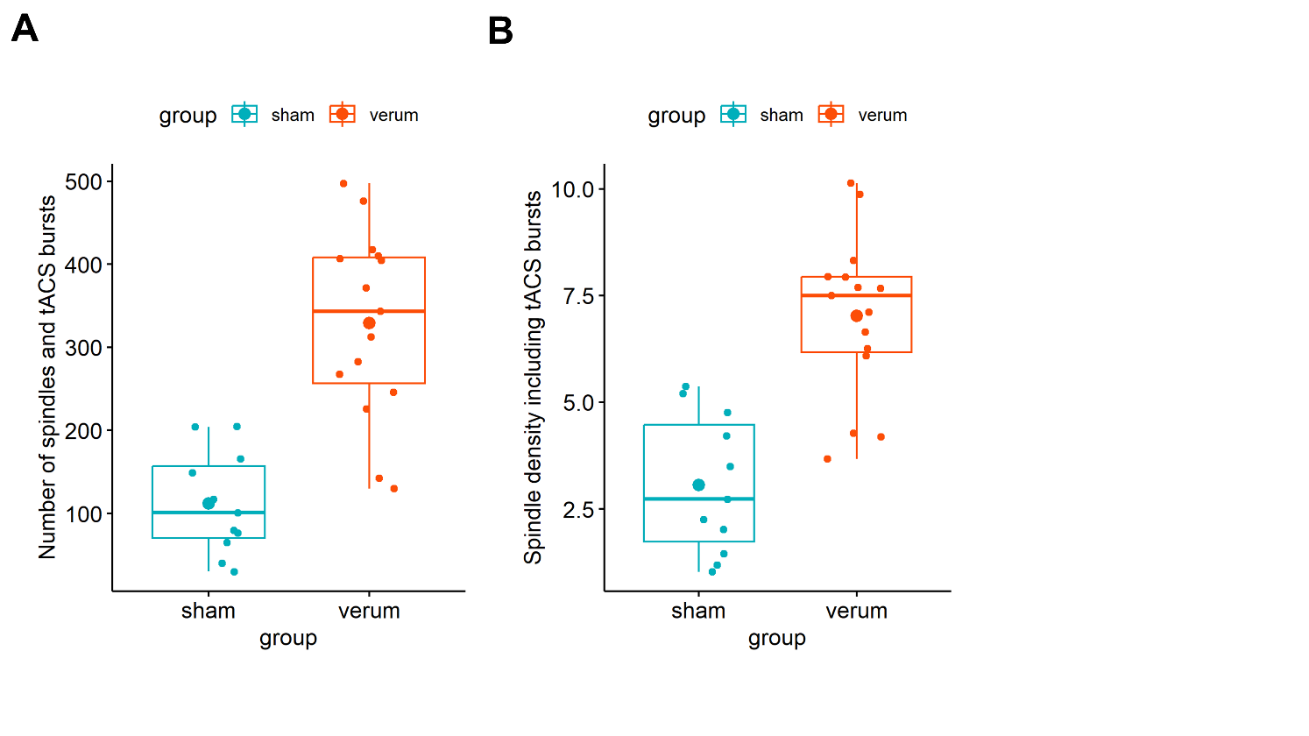
**

**Figure S2. Spindles and applied tACS bursts during the nap.** The blue boxplot depicts the sham group (N = 11), and the red depicts the verum group (N = 15*).* (**A**) The number of tACS bursts and spindles is 3 times higher in the verum group than the sham group (p < 0.001) resulting in (**B**) spindle density including the tACS bursts per min of NREM sleep (7.02 sp/min and 3.06 sp/min, respectively, p < 0.001). The spindle density including tACS bursts was doubled and, as expected, was larger in the verum group compared with the sham group. The box plots are with 75 and 25 percentiles. The horizontal lines in the box plots indicate the median values and the large, filled circles indicate the mean values. The small, filled circles are individual data points.

## Spindle data presented in the study

Fig. S3 shows the number of spindles (Fig. S3A), spindle density (Fig. S3B), and spindle duration (Fig. S3C), which did not differ between the sham and verum groups.

**
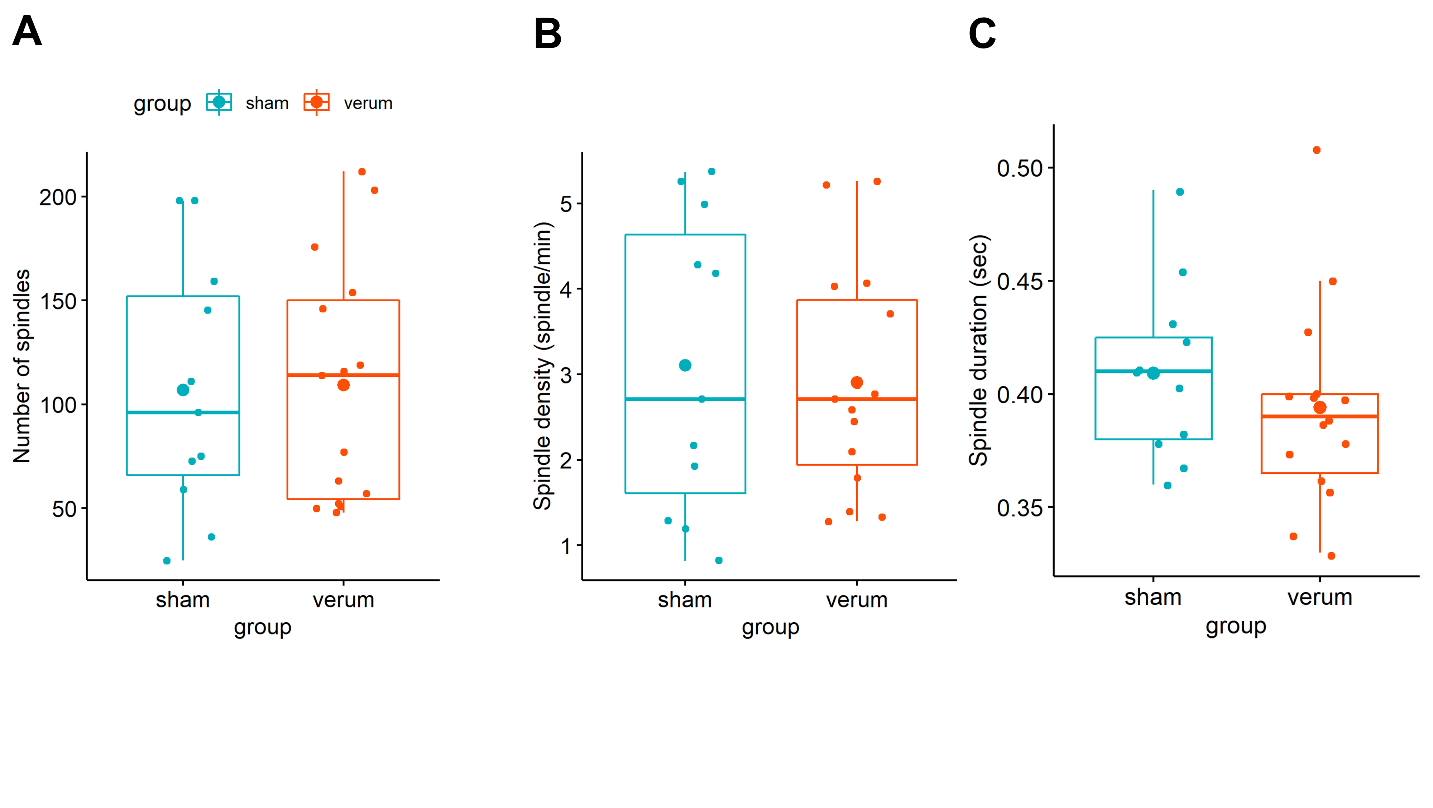
**

**Figure S3. The spindles were detected on the EEG trace during the nap.** (**A**) The number of spindles detected during sleep. (**B**) The density of spindles (number of spindles per minute of sleep). **(C)** The spindle duration. The blue boxplot depicts the sham group (N = 11), and the red depicts the verum group (N = 15)*.* The number of spindles, their density, and duration are similar in both groups. The number of spindles produced during the nap in sham and verum groups was similar (p = 0.92). The spindle density of the sham and the verum group was similar (p = 0.74). Also, the durations of spindles in the sham group and verum group did not differ (p = 0.36).

## NREM2 spindle association

We demonstrated that spindle density was associated with Overnight accuracy change in the sham group (Fig. 5B). As previous studies have demonstrated the importance of NREM2 spindles for memory consolidation (Nishida & Walker, 2007; Morin *et al.*, 2008; Lustenberger *et al.*, 2016), we performed the same analysis as the main analysis but only including NREM2 spindles (Fig. S4). The results demonstrated the same association, suggesting that our main finding was primarily driven by associations in NREM2.


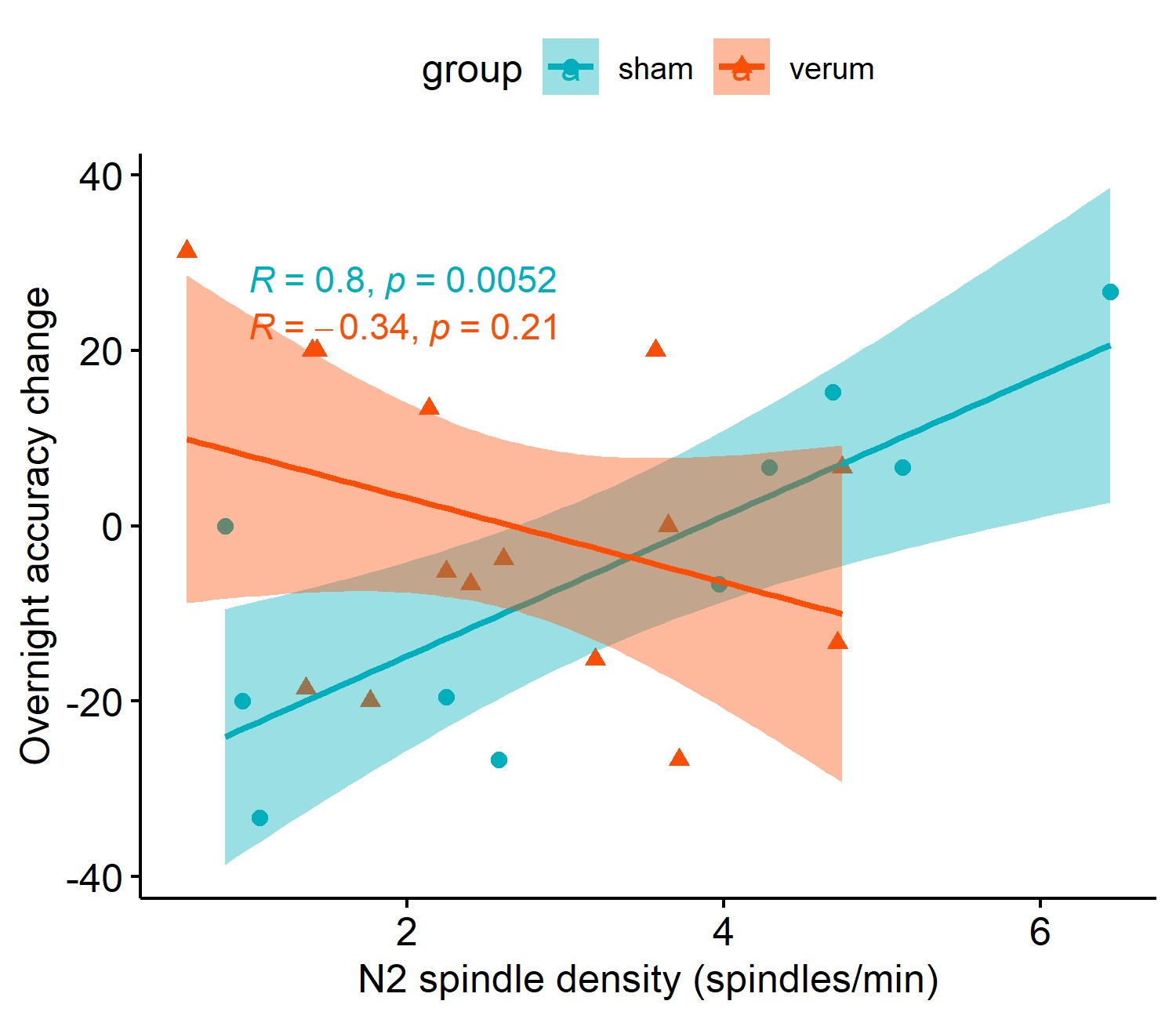


**Figure S4. Correlation between Overnight accuracy change and spindle density in NREM2.** The sham group is depicted in blue (N = 10) and the verum group is depicted in red (N = 15). The shaded areas depict 95% confidence intervals. For simplicity here in the supplementary information, linear model results including the factors Group, Spindle density, Group × Spindle density interaction term (same as in the Fig. 5B and Table 2) are not shown.

## Spindle organization

The temporal organization of spindles in train, also called cluster, has been described (Evans & Richardson, 1995; Nicolas *et al.*, 1997; Olbrich & Achermann, 2008) and recent studies demonstrated its importance for SDC (Antony *et al.*, 2018; Solano *et al.*, 2022). The present study was not designed to modulate spindle organization; however, results of spindle organization could be valuable to interested parties. We have investigated the aspect by extracting intervals between the start of a spindle and the start of the next spindle as inter-spindle intervals (ISIs) in NREM2. A train was defined as the minimum two spindles and subsequent spindles with ISI ≤ 6 s (Boutin *et al.*, 2018; Boutin & Doyon, 2020). The three metrics were extracted: number of spindle trains, number of spindles per train, and ISI per train, which did not differ between the two groups (number of spindle trains: p = 0.973; number of spindles per train: p = 0.352; ISI per train: p = 0.0774). The Fig. S5 shows associations of spindle organization metrics with Overnap or Overnight accuracy change. Overall, although results need to be interpreted cautiously due to a few factors related to the experimental design (i.e., artifactual 4 min time window and obscure time during the stimulation), there is no apparent behavioral association with these metrics nor differences between the sham and verum groups. The absence of spindle clustering contribution to SDC might be due to the age-related change of spindle organization. Since relatively few studies have examined this aspect of sleep, more research should investigate it with a dedicated experimental design.

**A B**


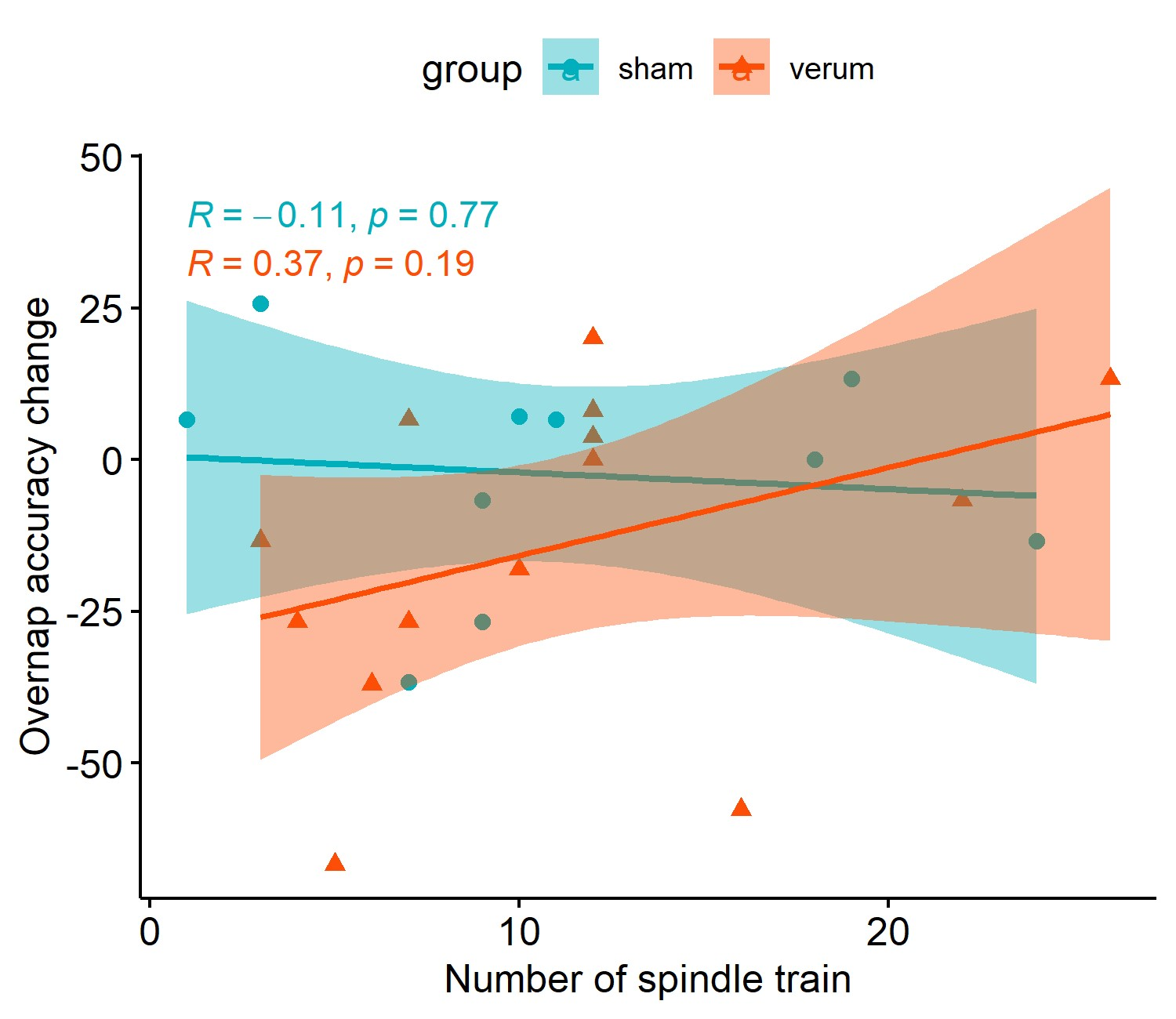

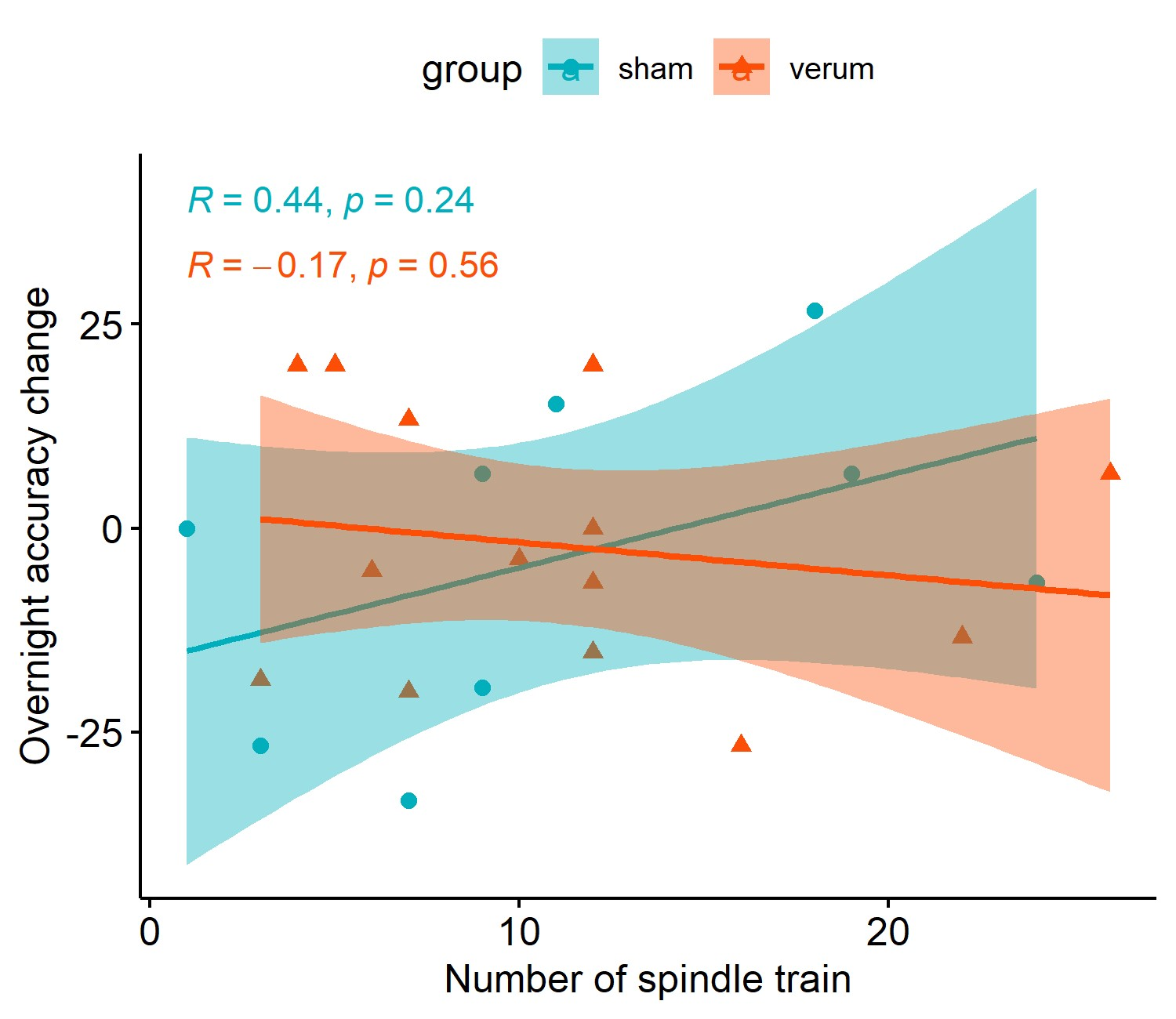


**C D**


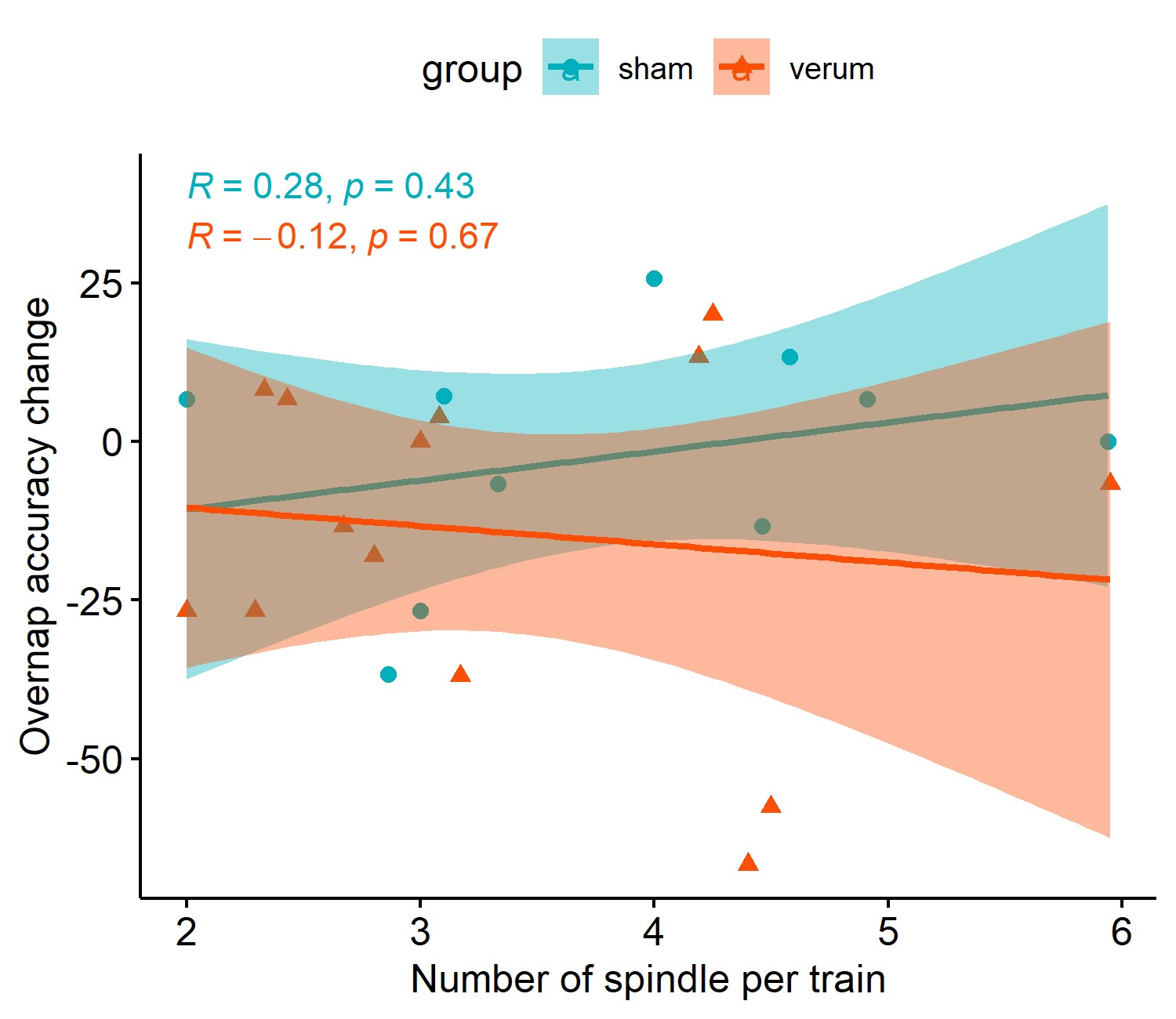

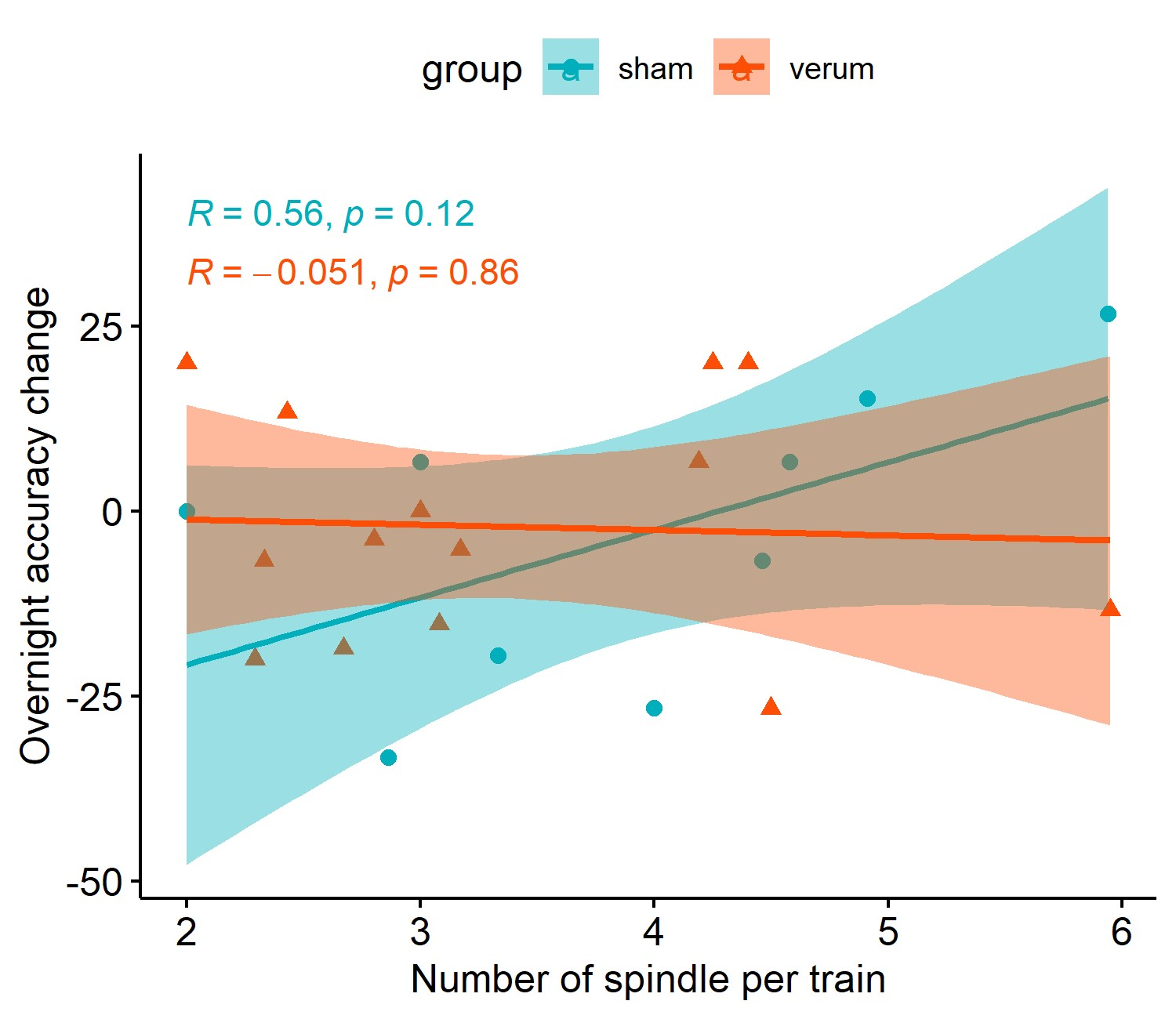


**E F**


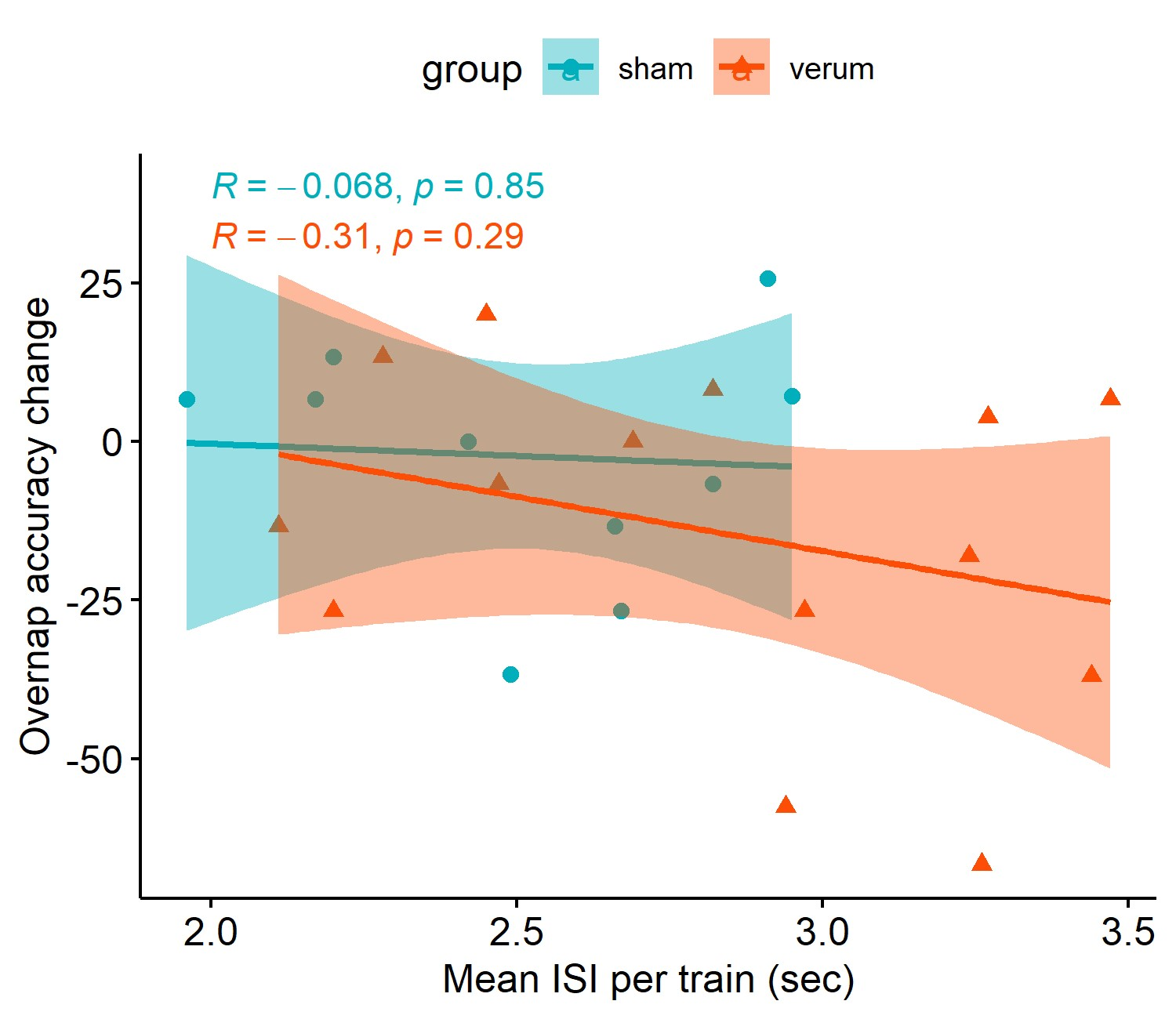

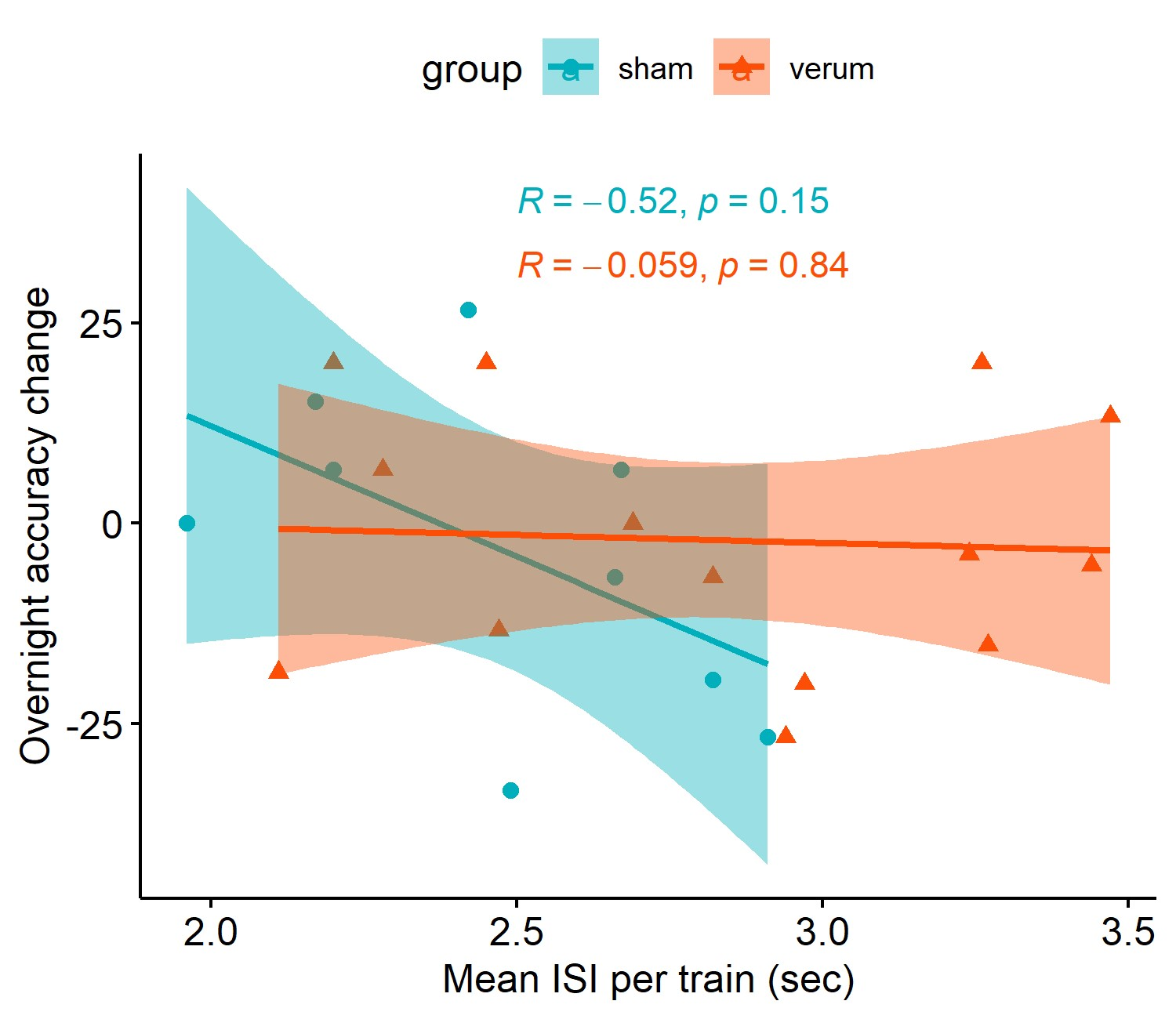


**Figure S5. Correlation between metrics of spindle organization and Overnap or Overnight accuracy change in NREM2.** (**A**) Overnap accuracy change and number of spindle train. (**B**) Overnight accuracy change and number of spindle train. (**C**) Overnap accuracy change and number of spindle per train, (**D**) Overnight accuracy change and number of spindle per train. (**E**) Overnap accuracy change and mean ISI per train. (**F**) Overnight accuracy change and mean ISI per train. The sham group is depicted in blue (overnap: N = 11; overnight: N = 10) and the verum group is depicted in red (N = 15). The shaded areas depict 95% confidence intervals. For simplicity here in the supplementary information, linear model results including the factors Group, Spindle density, Group × Spindle density interaction term (same as in the Fig. 5B and Table 2) are not shown.

# **Effects of spindle-inspired tACS on Overnap speed change and Overnight speed change**

The speed was measured from the onset of cursor movement at the baseline to the stop of cursor movement at a target bar in successful trials or outside of a target bar (continued movement pause for at least 200 ms) in unsuccessful trials. Then, the speed of successful and unsuccessful trials is averaged per block. Lower values mean better performance. Spindle density was not associated with Overnap speed accuracy and overnight speed accuracy. Thus, the spindle density was uniquely associated with 1) accuracy measure of behavior and 2) in the sham group.

## Overnap speed change

We compared the block before the nap (pre-nap speed) to the block after the nap (post-nap speed) and tested if Overnap speed change differed between groups (Fig. S6A). The effect of Time was significant (F_(1, 26)_ = 10.40, p = 0.003) but the effect of Group was not significant (F_(1, 26)_ = 1.64, p = 0.212). Additionally, the interaction term Time × Group was also not significant (F_(1, 26)_ = 0.18 p = 0.77), showing that the nap augmented the motor execution speed and thus diminished the motor performance in both groups.

## Overnight speed change

We compared the block before the night (pre-night speed) to the block after the night (post-night speed) and tested if overnight speed change differed between the groups (Fig. S6B). The effect of Time was significant (F_(1, 26)_ = 16.98, p = 0.0003) while the effect of Group was not significant (F_(1, 26)_ = 0.18, p = 0.76). Additionally, the interaction term Time × Group was also not significant (F_(1, 26)_ = 0.25, p = 0.62), suggesting that sleeping during the night elongated the speed and thus diminished motor performance similarly for both groups.


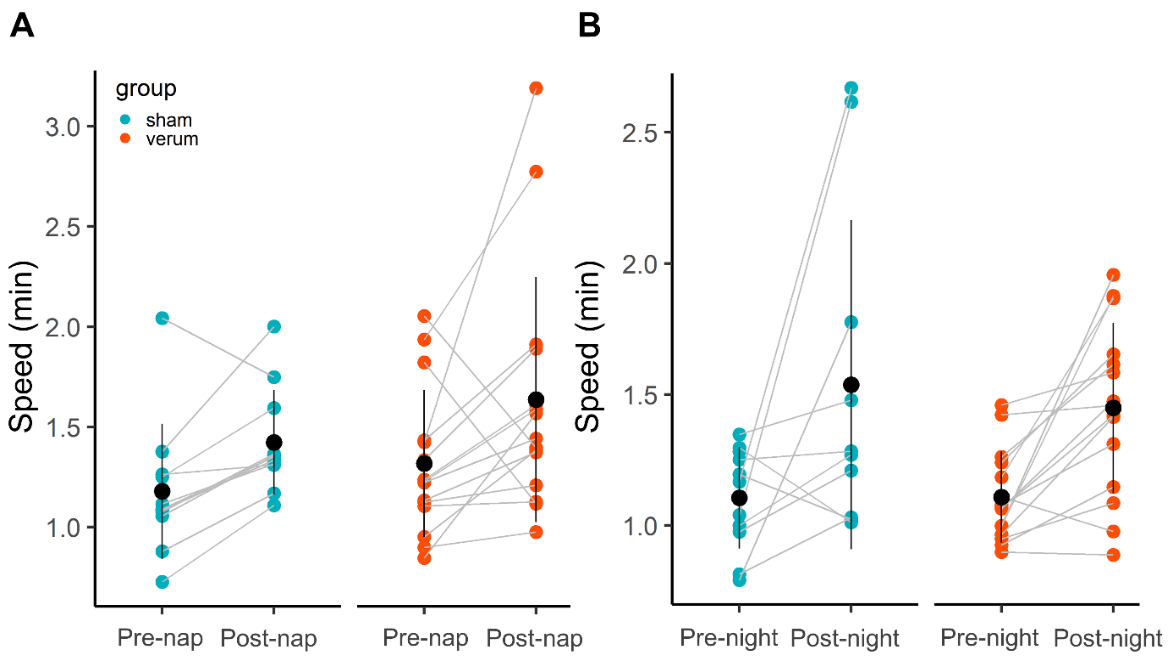


**Figure S6. Effect of spindle-inspired tACS on** **Overnap speed change and Overnight speed change.** (**A**) Overnap speed change: pre-nap and post-nap speed. (**B**) Overnight speed change: pre-night and post-night speed. The blue circles depict the sham group (pre-post nap: N = 11, pre-post night: N = 10) and the red depicts the verum group (N=15). The black circles indicate the mean values, and the vertical lines are the standard deviations. Sleep elongated motor execution speed and spindle-inspired tACS did not modulate Overnap speed change and overnight speed change.

# **Further analysis of Overnap speed change and Overnight speed change: effect of spindles**

## Effect of spindles on Overnap speed change

Linear model analysis did not reveal a significant main effect of Group (p = 0.82), Spindle density (p = 0.52) and Group × Spindle density interaction (p = 0.32, Fig. S7A).

## Effect of spindles on Overnight speed change

Linear model analysis did not reveal a significant main effect of Group (p = 0.54), Spindle density (p = 0.10) and Group × Spindle density interaction (p = 0.53, Fig. S7B).

*
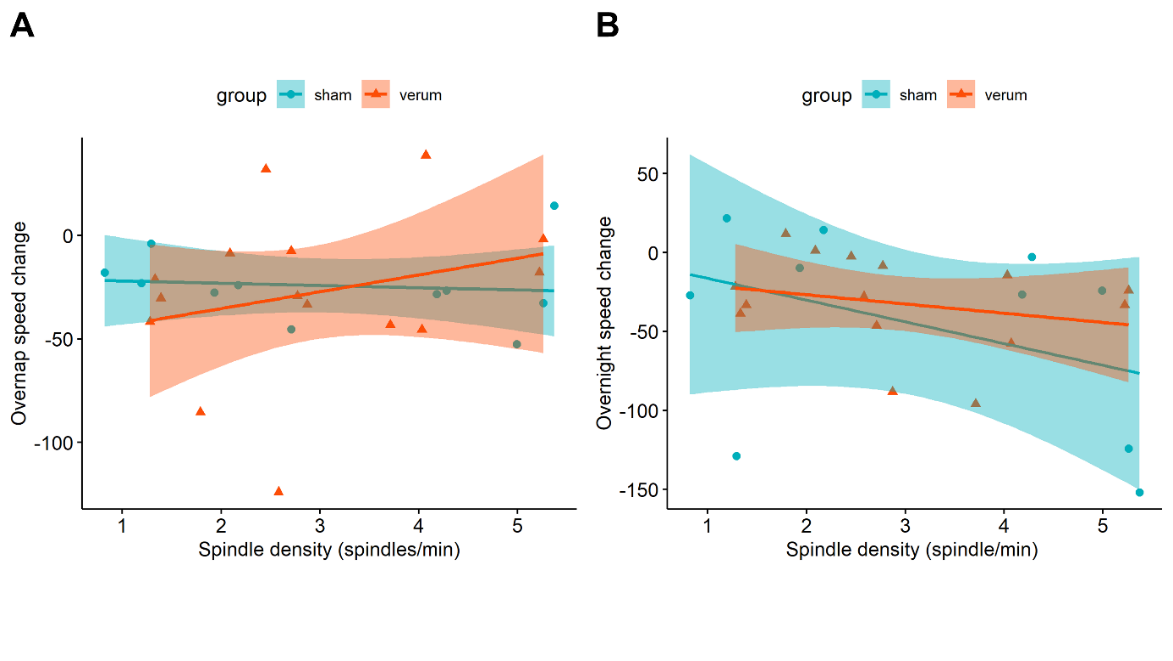
*

**Figure S7. Spindle association with Overnap speed change and Overnight speed change in both groups.** (**A**) Associations between spindle density and Overnap speed change. (**B**) Associations between spindle density and Overnight speed change. The blue lines depict the sham group (overnap: N = 11; overnight: N = 10) and the red lines depict the verum group (N = 15). The spindle density is the number of spindles per minute of NREM sleep during the nap (see Fig. S3). The shaded areas depict 95% confidence intervals. The statistical analysis was conducted with a linear model including factor group, spindles, and group × spindles interaction term. In both groups, the spindle density does not show a correlation with Overnap speed change and Overnight speed change. Contrary to Overnight accuracy change (Fig. 5B), spindles do not support Overnight speed change, suggesting a specific learning role of spindles oriented towards correct task execution (accuracy) rather than improving the speed of correct and incorrect trials.

# **Spindle-inspired tACS-related sensations during the nap**

**Verum versus sham distinction**. Participants could not effectively distinguish whether they received the verum or the sham stimulation during the nap. The proportion of correct distinctions was 0.27 and was not significantly different from the chance level p =1 and did not differ between verum and sham groups: χ2(1,26) = 0.001, p = 0.97).

**Reported sensations.** After the nap, the participants were asked about their perceived sensations during the stimulation. We applied a structured questionnaire, adapted from Antal and colleagues (Antal *et al.*, 2017) to check for the following sensations: itching, pain, burning, metallic/iron taste in the mouth, warmth, fatigue, and other types. Response options were: “0” = none, “1” = mild, “2” = moderate, “3” = strong to define the intensity of the sensation. Most of the participants did not report any stimulation sensations during the nap in the verum group (77.7%), and in the sham group (72.7%). Among a few participants who felt the stimulation, only mild sensations were reported in the verum group (5/15, 33.3%, tingling, and warm sensations) and in the sham group (3/11, 27.3%, tingling, warm and itching sensations). None of the reported sensations differed between verum and sham types of stimulation, please see the table below.

**Table S1.** Spindle-inspired tACS-related sensations for the verum and sham stimulation.

|  | **None** | | **Mild** | | **Moderate** | | **Strong** | | **Statistics** | |
| --- | --- | --- | --- | --- | --- | --- | --- | --- | --- | --- |
|  | **Verum** | **Sham** | **Verum** | **Sham** | **Verum** | **Sham** | **Verum** | **Sham** | **Chi-square** | |
| Itching | 100 | 90.9 | 0 | 9.1 | 0 | 0 | 0 | 0 | χ2 =1.4, p = 0.23 | |
| Pain | 100 | 100 | 0 | 0 | 0 | 0 | 0 | 0 | n/a |  |
| Burning | 100 | 100 | 0 | 0 | 0 | 0 | 0 | 0 | n/a |  |
| Warmth | 86.7 | 90.9 | 13.3 | 9.1 | 0 | 0 | 0 | 0 | χ2 = 0.1, p = 0.74 | |
| Metallic/iron taste | 100 | 100 | 0 | 0 | 0 | 0 | 0 | 0 | n/a |  |
| Fatigue | 100 | 100 | 0 | 0 | 0 | 0 | 0 | 0 | n/a |  |
| Others | 80 | 90.9 | 20 | 9.1 | 0 | 0 | 0 | 0 | χ2 = 2.3, p = 0.13 | |
| Values represent the percentage of participants who chose the intensity of the verum and sham stimulation sensation.  Abbreviations: Not applicable (n/a). A chi-squared test (with chisq.test of r) is performed to estimate the distribution of the variables between sham and verum groups. | | | | | | | | | | |

**Table S2.** Spindle-inspired tACS in the verum and sham groups.

|  | **Sham** | **Verum** |  |
| --- | --- | --- | --- |
|  | Mean ± SD | Mean ± SD | Statistics |
| **Number of blocks applied** |  |  |  |
| 6 stimulation blocks | 6 | 7 | - |
| 5 stimulation blocks | 2 | 2 | - |
| 4 stimulation blocks | 2 | 0 | - |
| 3 stimulation blocks | 0 | 3 | - |
| 2 stimulation blocks | 0 | 2 | - |
| 1 stimulation block | 1 | 1 | - |
| Number of blocks | 4.4 ± 1.8 | 5.0 ± 1.5 | F = 0.76 |
| Stimulation time (%) | 25.8 ± 9.1 | 24.4 ± 11.0 | F = 0.11 |
| Sleep/stimulation ratio | 58.5 ± 24.9 | 43.6 ± 20.8 | F = 2.77 |
| Total block duration (min) | 18.5 ± 5.8 | 16.5 ± 6.9 | F = 0.63 |

Abbreviations: standard deviation (SD), Chi-square (χ2), the stimulation time (%) is relative to TIB. Group F-tests are performed with *sumtable* function. Statistical significance markers: * p < 0.05; ** p < 0.01.

**Table S3.** Demographic data and questionnaires in the verum and sham groups.

|  | **Sham** | **Verum** |  |
| --- | --- | --- | --- |
|  | Mean ± SD | Mean ± SD | Statistics |
| **Demographic** |  |  |  |
| Age | 68.9 ± 3.5 | 70.5 ± 4.3 | F = 0.97 |
| Gender | M: 9.1% | M: 53.3% | χ2 = 5.5 |
| Body Mass Index | 23.5 ± 2.8 | 25.6 ± 4.1 | F = 1.97 |
| Level of Education | 3.4 ± 2.9 | 3.0 ± 2.8 | F = 0.01 |
| Physical Activity | 7.8 ± 3.2 | 6.9 ± 5.4 | F = 0.21 |
| **Questionnaires** |  |  |  |
| MoCA | 25.8 ± 2.9 | 26.0 ± 2.8 | F = 0.05 |
| PSQI | 4.9 ± 3.3 | 4.7 ± 2.2 | F = 0.05 |
| SSS-pre-nap | 2.2 ± 0.9 | 2.1 ± 0.9 | F = 0.09 |
| SSS-post-nap retest | 1.7 ± 1.1 | 2.1 ± 0.7 | F = 1.26 |
| SSS-post-night retest | 1.4 ± 0.7 | 1.9 ± 0.1 | F = 2.26 |
| **Motor assessments (left hand)** |  |  |  |
| Nine-hole peg test (sec) | 21.5 ± 2.9 | 22.9 ± 3.2 | F = 1.36 |
| Maximal grip force (Fist, newton) | 23.8 ± 8.4 | 33.6 ± 7.8 | F = 9.37** |
| Maximal grip force (Pinch, newton) | 3.7 ± 0.9 | 4.4 ± 0.9 | F = 3.13 |
| Maximal grip force (Key, newton) | 7.1 ± 1.0 | 8.2 ± 2.1 | F = 2.20 |

Abbreviations: standard deviation (sd), men (M), Chi-square (χ2), Group F-tests are performed with *sumtable* function of r. A chi-squared test (with chisq. test of r) is performed to estimate the distribution of the variables between sham and verum groups. Statistical significance markers: * p < 0.05; ** p < 0.01.

# **References**

Antal A et al. (2017). Low intensity transcranial electric stimulation: Safety, ethical, legal regulatory and application guidelines. *Clin Neurophysiol* **128,** 1774–1809.

Antony JW, Piloto L, Wang M, Pacheco P, Norman KA & Paller KA (2018). Sleep Spindle Refractoriness Segregates Periods of Memory Reactivation. *Curr Biol* **28,** 1736-1743.e4.

Backhaus W, Braass H, Renné T, Gerloff C & Hummel FC (2016*a*). Motor Performance Is not Enhanced by Daytime Naps in Older Adults. *Front Aging Neurosci* **8,** 125.

Backhaus W, Braaß H, Renné T, Krüger C, Gerloff C & Hummel FC (2016*b*). Daytime sleep has no effect on the time course of motor sequence and visuomotor adaptation learning. *Neurobiol Learn Mem* **131,** 147–154.

Backhaus W, Kempe S & Hummel FC (2016*c*). The effect of sleep on motor learning in the aging and stroke population–a systematic review. *Restor Neurol Neurosci* **34,** 153–164.

Boutin A & Doyon J (2020). A sleep spindle framework for motor memory consolidation. *Philos Trans R Soc B Biol Sci* **375,** 20190232.

Boutin A, Pinsard B, Boré A, Carrier J, Fogel SM & Doyon J (2018). Transient synchronization of hippocampo-striato-thalamo-cortical networks during sleep spindle oscillations induces motor memory consolidation. *NeuroImage* **169,** 419–430.

Debarnot U, Castellani E, Valenza G, Sebastiani L & Guillot A (2011). Daytime naps improve motor imagery learning. *Cogn Affect Behav Neurosci* **11,** 541–550.

Doyon J, Korman M, Morin A, Dostie V, Tahar AH, Benali H, Karni A, Ungerleider LG & Carrier J (2009). Contribution of night and day sleep vs. simple passage of time to the consolidation of motor sequence and visuomotor adaptation learning. *Exp Brain Res* **195,** 15–26.

Evans BM & Richardson NE (1995). Demonstration pf a 3–5s periodicity between the spindle bursts in NREM sleep in man. *J Sleep Res* **4,** 196–197.

Fang Z, Smith DM, Albouy G, King BR, Vien C, Benali H, Carrier J, Doyon J & Fogel S (2021). Differential Effects of a Nap on Motor Sequence Learning-Related Functional Connectivity Between Young and Older Adults. *Front Aging Neurosci* **13,** 747358.

Fogel SM, Albouy G, Vien C, Popovicci R, King BR, Hoge R, Jbabdi S, Benali H, Karni A & Maquet P (2014). fMRI and sleep correlates of the age‐related impairment in motor memory consolidation. *Hum Brain Mapp* **35,** 3625–3645.

Gudberg C, Wulff K & Johansen-Berg H (2015). Sleep-dependent motor memory consolidation in older adults depends on task demands. *Neurobiol Aging* **36,** 1409–1416.

Gui W-J, Li H-J, Guo Y-H, Peng P, Lei X & Yu J (2017). Age-related differences in sleep-based memory consolidation: A meta-analysis. *Neuropsychologia* **97,** 46–55.

King BR, Hoedlmoser K, Hirschauer F, Dolfen N & Albouy G (2017). Sleeping on the motor engram: the multifaceted nature of sleep-related motor memory consolidation. *Neurosci Biobehav Rev* **80,** 1–22.

King BR, Saucier P, Albouy G, Fogel SM, Rumpf J-J, Klann J, Buccino G, Binkofski F, Classen J, Karni A & Doyon J (2016). Cerebral Activation During Initial Motor Learning Forecasts Subsequent Sleep-Facilitated Memory Consolidation in Older Adults. *Cereb Cortex*bhv347.

Korman M, Dagan Y & Karni A (2015). Nap it or leave it in the elderly: A nap after practice relaxes age-related limitations in procedural memory consolidation. *Neurosci Lett* **606,** 173–176.

Lustenberger C, Boyle MR, Alagapan S, Mellin JM, Vaughn BV & Fröhlich F (2016). Feedback-Controlled Transcranial Alternating Current Stimulation Reveals a Functional Role of Sleep Spindles in Motor Memory Consolidation. *Curr Biol* **26,** 2127–2136.

Maier JG, Piosczyk H, Holz J, Landmann N, Deschler C, Frase L, Kuhn M, Klöppel S, Spiegelhalder K & Sterr A (2017). Brief periods of NREM sleep do not promote early offline gains but subsequent on-task performance in motor skill learning. *Neurobiol Learn Mem* **145,** 18–27.

Mander BA, Winer JR & Walker MP (2017*a*). Sleep and Human Aging. *Neuron* **94,** 19–36.

Mander BA, Zhu AH, Lindquist JR, Villeneuve S, Rao V, Lu B, Saletin JM, Ancoli-Israel S, Jagust WJ & Walker MP (2017*b*). White matter structure in older adults moderates the benefit of sleep spindles on motor memory consolidation. *J Neurosci* **37,** 11675–11687.

Morin A, Doyon J, Dostie V, Barakat M, Tahar AH, Korman M, Benali H, Karni A, Ungerleider LG & Carrier J (2008). Motor Sequence Learning Increases Sleep Spindles and Fast Frequencies in Post-Training Sleep. *Sleep* **31,** 1149–1156.

Nicolas A, Rompré S, Dumont M, Laberge L & Montplaisir J (1997). Four to five seconds periodicity of sleep spindles in different age groups. *Sleep Res* **26,** 31.

Nishida M & Walker MP (2007). Daytime naps, motor memory consolidation and regionally specific sleep spindles. *PloS One* **2,** e341.

Olbrich E & Achermann P (2008). Analysis of the Temporal Organization of Sleep Spindles in the Human Sleep EEG Using a Phenomenological Modeling Approach. *J Biol Phys* **34,** 241–249.

Pan SC & Rickard TC (2015). Sleep and motor learning: is there room for consolidation? *Psychol Bull* **141,** 812.

Robertson EM, Pascual-Leone A & Miall RC (2004). Current concepts in procedural consolidation. *Nat Rev Neurosci* **5,** 576.

Schmid D, Erlacher D, Klostermann A, Kredel R & Hossner E-J (2020). Sleep-dependent motor memory consolidation in healthy adults: A meta-analysis. *Neurosci Biobehav Rev* **118,** 270–281.

Solano A, Riquelme LA, Perez-Chada D & Della-Maggiore V (2022). Visuomotor Adaptation Modulates the Clustering of Sleep Spindles Into Trains. *Front Neurosci*; DOI: 10.3389/fnins.2022.803387.

Tucker M, McKinley S & Stickgold R (2011). Sleep Optimizes Motor Skill in Older Adults: SLEEP AND MOTOR MEMORY IN OLDER ADULTS. *J Am Geriatr Soc* **59,** 603–609.

Vien C, Boré A, Lungu O, Benali H, Carrier J, Fogel S & Doyon J (2016). Age-related white-matter correlates of motor sequence learning and consolidation. *Neurobiol Aging* **48,** 13–22.

Walker MP (2005). A refined model of sleep and the time course of memory formation. *Behav Brain Sci* **28,** 51–64.

Walker MP, Brakefield T, Morgan A, Hobson JA & Stickgold R (2002). Practice with sleep makes perfect: sleep-dependent motor skill learning. *Neuron* **35,** 205–211.

Walker MP & Stickgold R (2004). Sleep-dependent learning and memory consolidation. *Neuron* **44,** 121–133.

# **Figure and table caption**

**Figure S1. Effect of napping on Overnap accuracy change and Overnight accuracy change.** (**A**) Overnap accuracy change. (**B**) Overnight accuracy change. The dark blue circles depict the sham group (nap group) (overnap: N = 11; overnight: N = 10) and the dark red circles depict the no-nap group (N = 10). The block circles indicate the mean values, and the vertical lines are the standard deviation. Overnap accuracy change and Overnight accuracy changes were similar in both groups. Napping did not modulate memory consolidation.

**Figure S2. Spindles and applied tACS bursts during the nap.** The blue boxplot depicts the sham group (N = 11), and the red depicts the verum group (N = 15*).* (**A**) The number of tACS bursts and spindles is 3 times higher in the verum group than the sham group (p < 0.001) resulting in (**B**) spindle density including the tACS bursts per min of NREM sleep (7.02 sp/min and 3.06 sp/min, respectively, p < 0.001). The spindle density including tACS bursts was doubled and, as expected, was larger in the verum group compared with the sham group. The box plots are with 75 and 25 percentiles. The horizontal lines in the box plots indicate the median values and the large, filled circles indicate the mean values. The small, filled circles are individual data points.

**Figure S3. The spindles were detected on the EEG trace during the nap.** (**A**) The number of spindles detected during sleep. (**B**) The density of spindles (number of spindles per minute of sleep). (**C**) The spindle duration. The blue boxplot depicts the sham group (N = 11), and the red depicts the verum group (N = 15)*.* The number of spindles, their density, and duration are similar in both groups. The number of spindles produced during the nap in sham and verum groups was similar (p = 0.92). The spindle density of the sham and the verum group was similar (p = 0.74). Also, the durations of spindles in the sham group and verum group did not differ (p = 0.36).

**Figure S4. Correlation between Overnight accuracy change and spindle density in NREM2.** The sham group is depicted in blue (N = 10) and the verum group is depicted in red (N = 15). The shaded areas depict 95% confidence intervals. For simplicity here in the supplementary information, linear model results including the factors Group, Spindle density, Group × Spindle density interaction term (same as in the Fig. 5B and Table 2) are not shown.

**Figure S5. Correlation between metrics of spindle organization and Overnap or Overnight accuracy change in NREM2.** (**A**) Overnap accuracy change and number of spindle train. (**B**) Overnight accuracy change and number of spindle train. (**C**) Overnap accuracy change and number of spindle per train, (**D**) Overnight accuracy change and number of spindle per train. (**E**) Overnap accuracy change and mean ISI per train. (**F**) Overnight accuracy change and mean ISI per train. The sham group is depicted in blue (overnap: N = 11; overnight: N = 10) and the verum group is depicted in red (N = 15). The shaded areas depict 95% confidence intervals. For simplicity here in the supplementary information, linear model results including the factors Group, Spindle density, Group × Spindle density interaction term (same as in the Fig. 5B and Table 2) are not shown.

**Figure S6. Effect of spindle-inspired tACS on Overnap speed change and Overnight speed change.** (**A**) Overnap speed change: pre-nap and post-nap speed. (**B**) Overnight speed change: pre-night and post-night speed. The blue circles depict the sham group (pre-post nap: N = 11, pre-post night: N = 10) and the red depicts the verum group (N=15). The black circles indicate the mean values, and the vertical lines are the standard deviations. Sleep elongated motor execution speed and spindle-inspired tACS did not modulate Overnap speed change and overnight speed change.

**Figure S7. Spindle association with Overnap speed change and Overnight speed change in both groups.** (**A**) Associations between spindle density and Overnap speed change. (**B**) Associations between spindle density and Overnight speed change. The blue lines depict the sham group (overnap: N = 11; overnight: N = 10) and the red lines depict the verum group (N = 15). The spindle density is the number of spindles per minute of NREM sleep during the nap (see Fig. S3). The shaded areas depict 95% confidence intervals. The statistical analysis was conducted with a linear model including factor group, spindles, and group × spindles interaction term. In both groups, the spindle density does not show a correlation with Overnap speed change and Overnight speed change. Contrary to Overnight accuracy change (Fig. 5B), spindles do not support Overnight speed change, suggesting a specific learning role of spindles oriented towards correct task execution (accuracy) rather than improving the speed of correct and incorrect trials.

**Table S1.** Spindle-inspired tACS-related sensations for the verum and sham stimulation.

**Table S2.** Spindle-inspired tACS in the verum and sham group.

**Table S3.** Demographic data and questionnaires in the verum and sham group.
